# Supplementary material for: Soluble urokinase plasminogen activator receptor levels predict survival in patients with portal hypertension undergoing TIPS
Source: JHEP Rep. 2024 Mar 4;6(5):101054. doi: 10.1016/j.jhepr.2024.101054 (PMC11053213; doi:10.1016/j.jhepr.2024.101054)
Supplement: Multimedia component 1 [file mmc1.pdf]

Soluble urokinase plasminogen activator receptor levels predict survival in patients with portal hypertension undergoing TIPS

Sven H. Loosen, Fabian Benz, Raphael Mohr, Philipp A. Reuken, Theresa H. Wirtz, Lioba Junker, Christian Jansen, Carsten Meyer, Michael Praktiknjo, Alexander Wree, Johanna Reißing, Münevver Demir, Mihael Vucur, Robert Schierwagen, Andreas Stallmach, Anselm Kunstein, Johannes Bode, Christian Trautwein, Frank Tacke, Tom Luedde, Tony Bruns, Jonel Trebicka, Christoph Roderburg

Table of contents

Supplementary Figures……………………………………………………………………..2

Supplementary Tables…………………………………………………………………8

Supplementary Figures

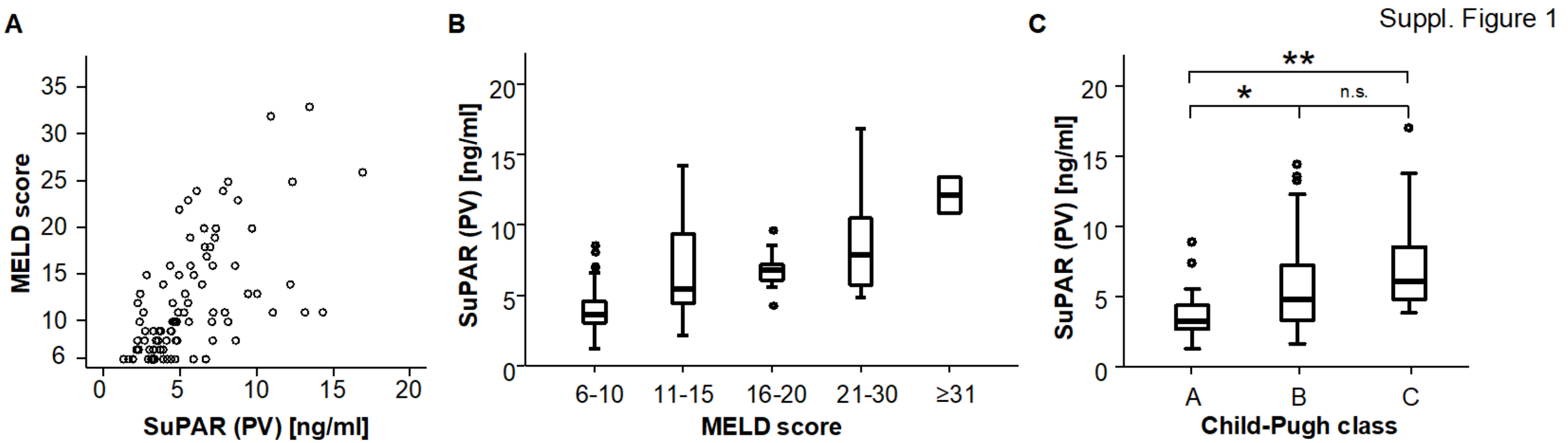

Fig. S1: (A) Portal vein (PV) suPAR levels correlate with the patients' MELD score. (B) PV suPAR concentrations show a stepwise increase in patients with more advanced MELD score. (C) PV suPAR concentrations show a significant and stepwise increase in patients with more advanced Child-Pugh class.

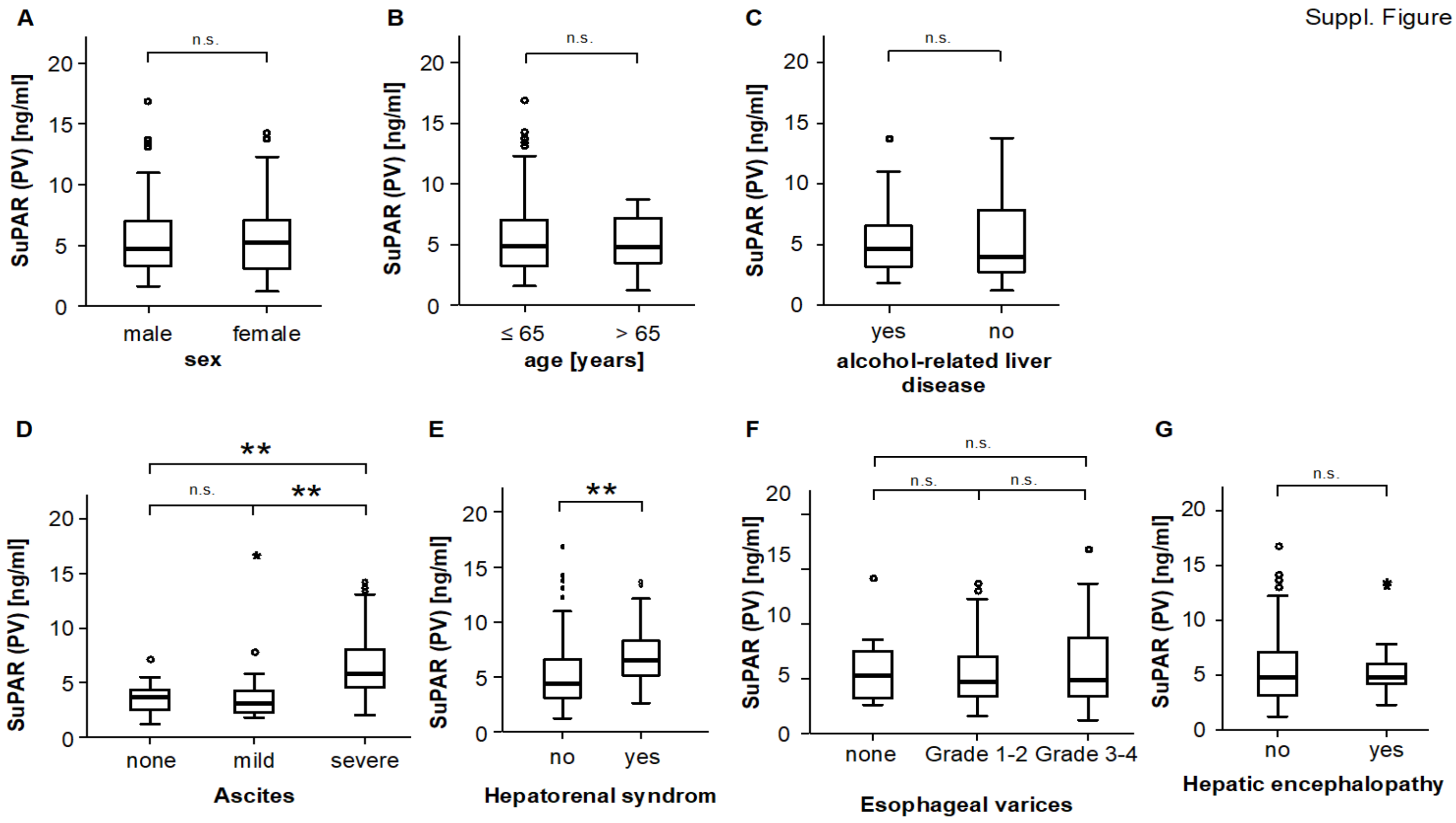

Fig. S2: (A-C) Hepatic vein (HV) suPAR levels are comparable in male and female patients, in patients younger or older than 65 years, and in patients with or without alcohol-related liver disease. (D) HV suPAR levels are significantly higher among patients with moderate to severe ascites compared to patients without or mild ascites. (E) HV suPAR levels are significantly higher in patients with hepatorenal syndrome (HRS) compared to patients with normal renal function. (F and G) Patients with or without esophageal varices or hepatic encephalopathy have comparable HV suPAR concentrations.

Suppl. Figure 3

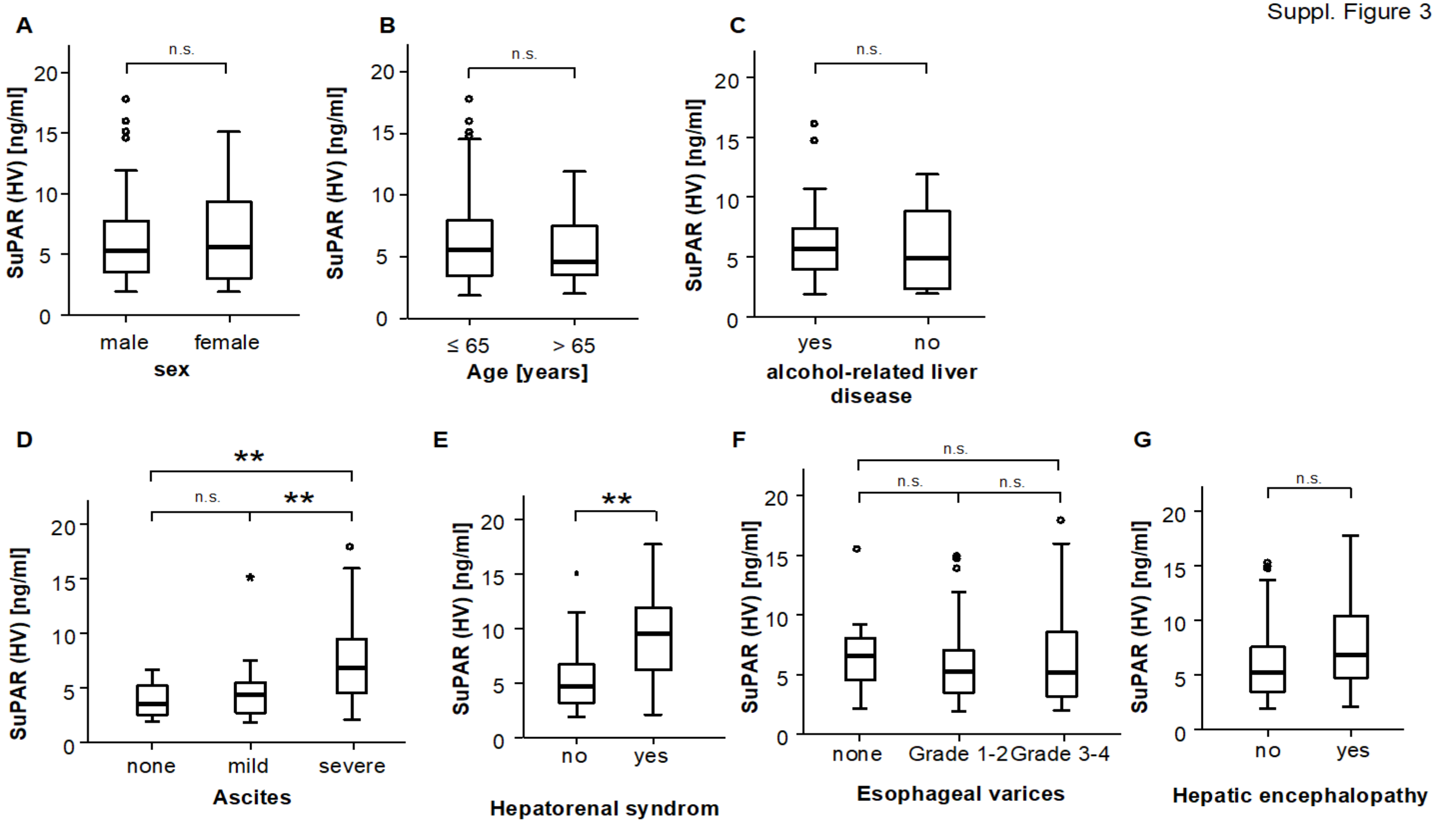

Fig. S3: (A-C) Portal vein (PV) suPAR levels are comparable in male and female patients, in patients younger or older than 65 years, and in patients with or without alcohol-related liver disease. (D) PV suPAR levels are significantly higher among patients with moderate to severe ascites compared to patients without or mild ascites. (E) PV suPAR levels are significantly higher in patients with hepatorenal syndrome (HRS) compared to patients with normal renal function. (F and G) Patients with or without esophageal varices or hepatic encephalopathy have comparable PV suPAR concentrations.

Supp. Figure 4

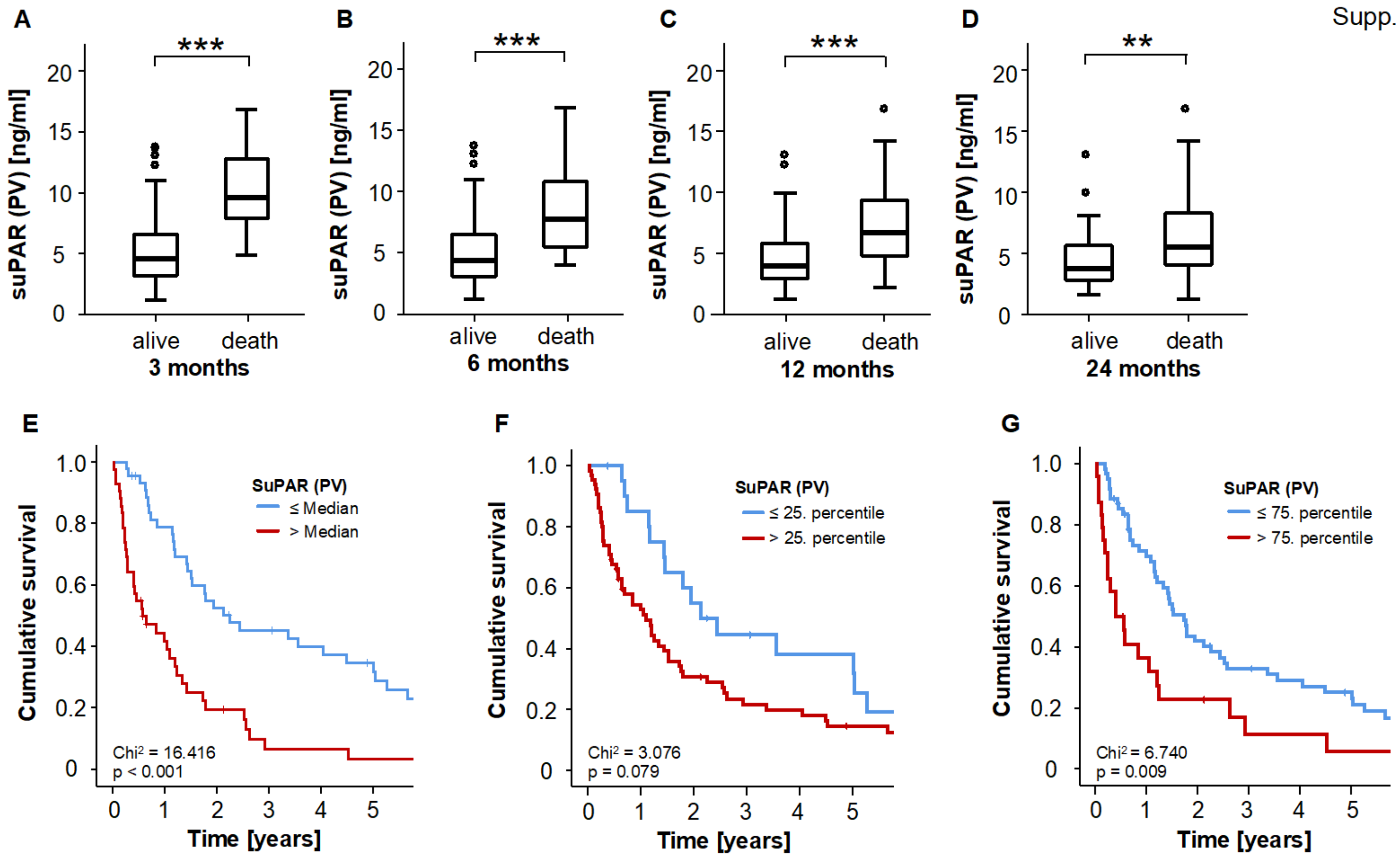

Fig. S4: Portal vein (PV) suPAR levels are significantly higher in patients who died within 3 (A), 6 (B), 12 (C) or 24 months (D) after TIPS insertion. The median OS is reduced in TIPS patients with baseline PV suPAR concentrations above the median (E) or the lower/upper quartile (F, G).

Suppl Figure 5

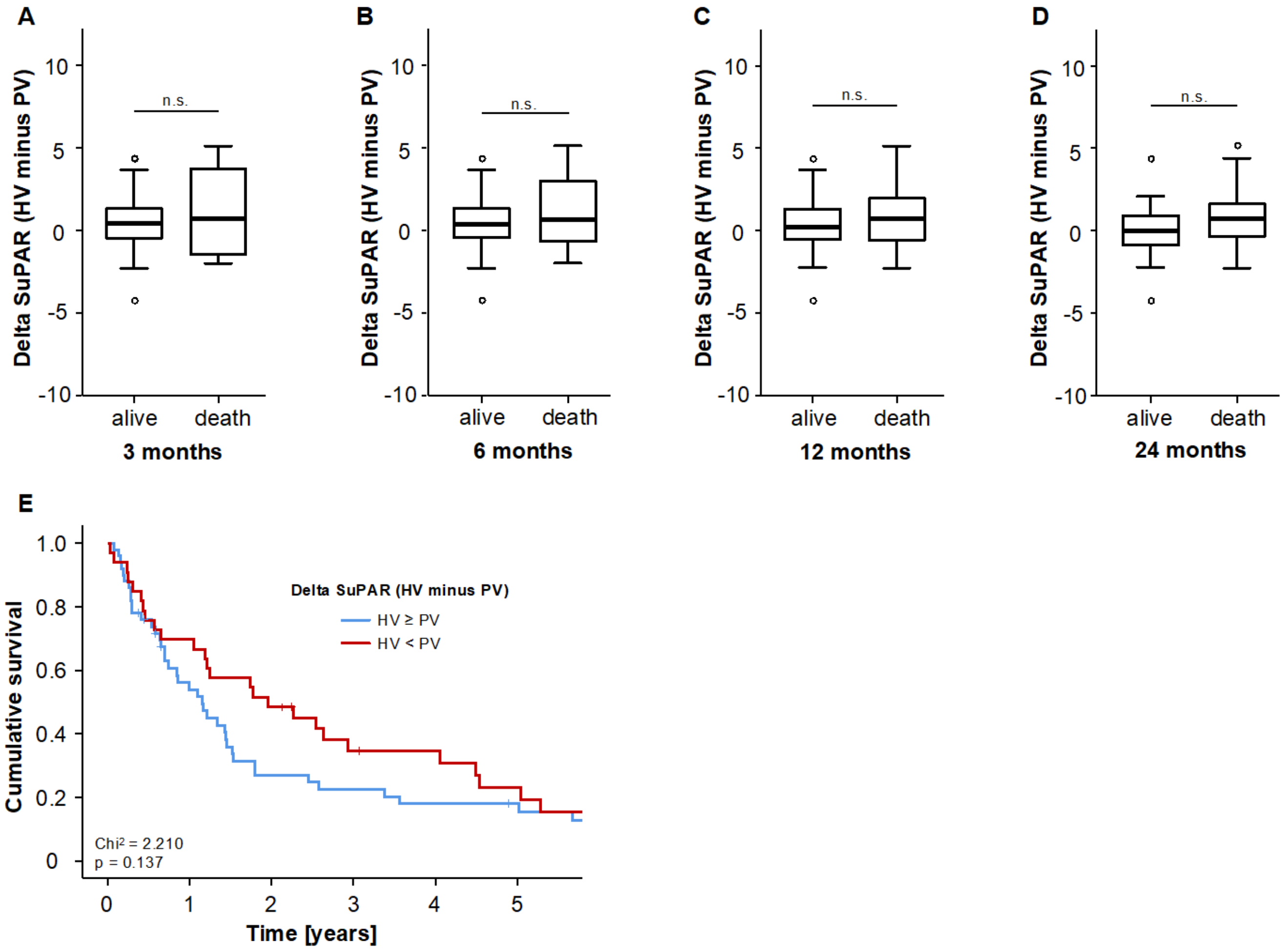

Fig. S5: (A-D) The individual ratio between HV and PV suPAR concentrations (delta suPAR = HV-PV) is unaltered TIPS patients who did or did not survive the 3-, 6-, 12-, or 24-months period following TIPS insertion. (E) There is no survival benefit for TIPS patients with either a positive or negative delta suPAR.

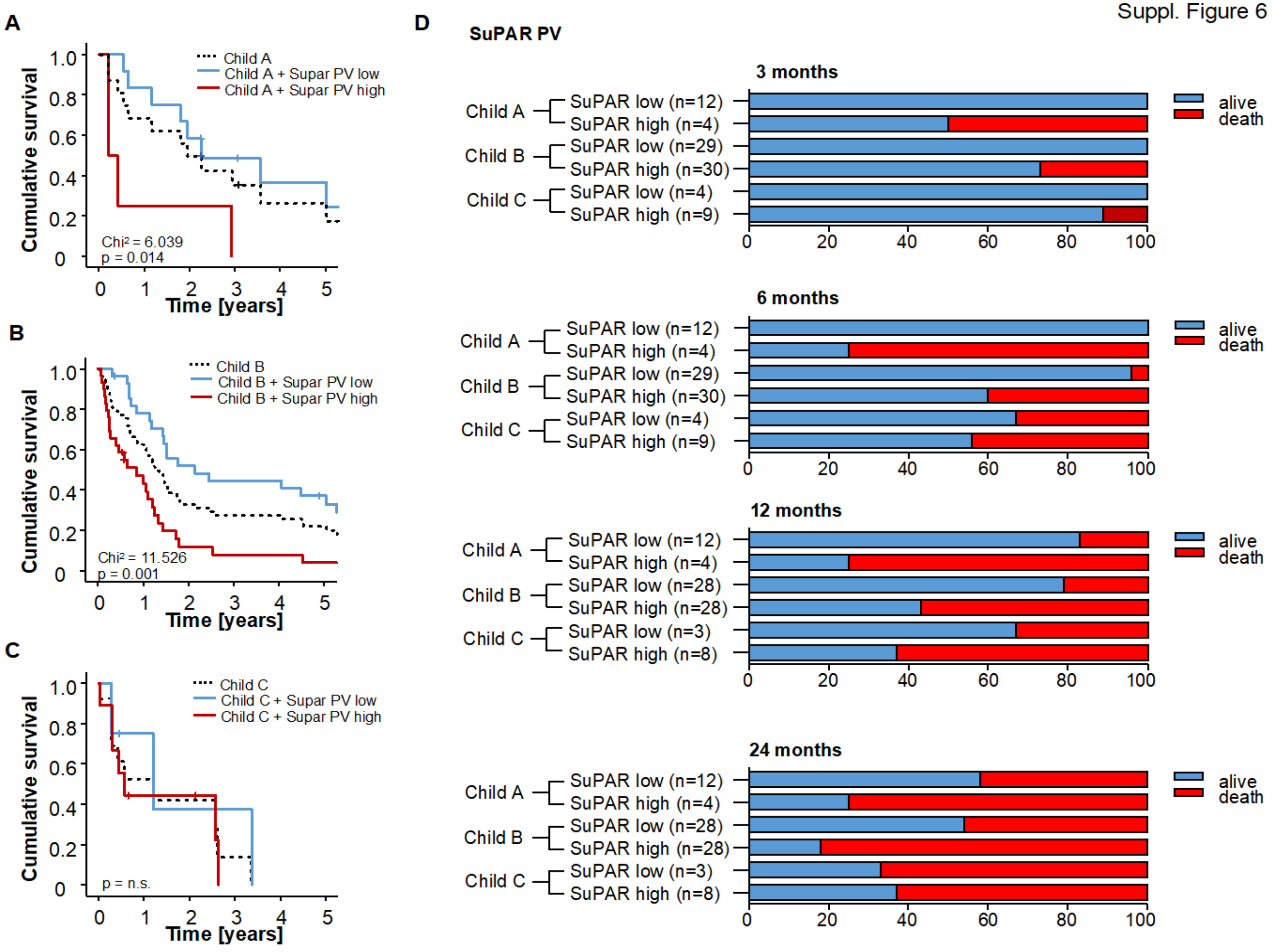

Fig. S6: (A) In Child A patients, elevated PV suPAR levels identify a subgroup of TIPS patients with a significantly impaired post-interventional median overall survival, which is lower compared to patients with Child B and Child C cirrhosis. (B) In Child B patients, elevated PV suPAR levels identify a subgroup of TIPS patients with a significantly impaired post-interventional outcome. (C) This finding is not observed in Child C patients. (D) Number of Child-Pugh stage-stratified deaths after 3, 6, 12, and 24 months in TIPS patient with high or low baseline PV suPAR levels.

Supplementary Tables

Table S1. Clinical and laboratory parameters of the training cohort.

	**SuPAR serum concentration[ng/ml]**		
**Parameters**	**Hepatic vein**	**Portal vein**	**p-value**
All patients (n=99)	5.27 (1.90-17.76)	4.76 (1.22-16.84)	p<0.01
Sex			
male: 67 %	5.27 (1.90-17.76)	4.70 (1.61-16.84)	p<0.05
female: 33 %	5.56 (1.92-15.10)	5.26 (1.22-14.23)	p<0.05
Age: 59 years (36-77)			
≤ 65 years: 78 %	5.56 (1.90-17.76)	4.85 (1.58-16.84)	p<0.05
> 65 years: 22 %	4.65 (2.05-11.91)	4.79 (1.22-8.70)	n.s.
BMI: 24.5 kg/m² (15.2-38.9)			
<20: 16 %	5.10 (1.92-11.46)	4.96 (1.58-13.75)	n.s.
20-25: 41 %	6.04 (1.99-14.56)	4.79 (1.22-10.85)	p<0.05
>25: 43 %	5.08 (1.90-15.96)	4.19 (1.83-13.67)	p<0.05
Etiology			
alcoholic: 73 %	5.70 (1.90-15.96)	3.95 (1.22-13.75)	p<0.05
non-alcoholic: 27 %	4.91 (1.92-11.91)	4.96 (1.58-13.75)	n.s.
Ascites			
no: 19 %	3.56 (1.92-6.65)	3.65 (1.22-7.19)	n.s.
mild: 17 %	4.39 (1.90-15.10)	3.09 (1.83-16.84)	p<0.05
moderate to severe: 64 %	6.86 (2.11-17.76)	5.81 (2.03-14.23)	p<0.05
History of hepatic			
encephalopathy	5.27 (1.90-15.10)	4.79 (1.22-16.84)	p<0.05
no: 85 %	6.16 (2.07-17.76)	4.79 (2.28-13.67)	p<0.05
yes: 15 %			
Hepatorenal syndrome			
no: 77 %	4.70 (1.90-15.10)	4.35 (1.22-16.84)	p<0.05
yes: 23 %	9.54 (2.11-17.76)	6.54 (2.61-13.67)	p<0.05
Esophageal varices			
no: 12 %	6.58 (2.11-15.10)	5.26 (2.61-14.23)	p<0.05
grade I-II: 66 %	5.27 (1.90-14.76)	4.70 (1.58-13.75)	p<0.05
grade III-IV: 22 %	5.15 (1.99-17.76)	4.86 (1.22-16.84)	n.s.
Child category			
Child A: 17 %	2.92 (1.92-9.54)	3.19 (1.22-8.70)	n.s.
Child B: 67 %	5.25 (1.90-17.76)	4.79 (1.61-14.23)	p<0.05
Child C: 16 %	7.34 (4.50-15.96)	6.04 (3.81-16.84)	p<0.05
MELD score: 10 (6-33)			
6-10: 52 %	4.39 (1.90-10.69)	3.67 (1.22-8.56)	p<0.05
11-15: 24 %	5.59 (2.60-15.10)	5.45 (2.16-14.23)	n.s.
16-20: 13 %	7.06 (4.50-14.76)	6.78 (4.27-9.62)	n.s.
21-30: 9%	9.96 (4.47-15.10)	7.89 (4.87-16.84)	n.s.
>30: 2%	16.16 (14.56-17.76)	12.11 (10.85-13.37)	n.s.

Data are shown as median and ranges

Table S2. Laboratory parameters (hepatic vein) of the training cohort.

Laboratory parameters	Value
Sodium	135 mmol/l (119-143)
Potassium	4.2 mmol/l (2.6-5.8)
Creatinine	1.1 mg/dl (0.5-8.2)
Urea	44 mg/dl (9-225)
Bilirubin	1.3 mg/dl (0.4-16.9)
Albumin	32 g/dl (11-56)
International Normalized Ratio (INR)	1.13 (0.95-2.40)
Partial thromboplastin time (PTT)	28 seconds (22-100)
Thrombocyte count	104 /nl (27-389)
Leukocyte count	5.5 /nl (1.4-22.3)
Glutamat-Oxalacetat-Transaminase (GOT)	21 U/l (8-73)
Glutamat-Pyruvat-Transaminase (GPT)	18 U/l (4-113)
Gamma-Glutamyltransferase (GGT)	57 U/l (8-1469)
Cholinesterase (ChE)	1804 U/l (282-11937)
Ammonia (NH3)	47 µmol/l (4-137)

Data are shown as median and ranges

Table S3. Hemodynamic parameters of the training cohort before TIPS.
Data are shown as median and ranges

Hemodynamic parameters before TIPS	Values
Mean arterial pressure	84 mmHg (48-129)
Central venous pressure	10 cmH2O (0-22)
Portal pressure	30 mmHg (18-49)
Hepatic venous pressure gradient (HVPG)	20 mmHg (11-35)
Portal venous velocity	16 (5-40)
Portal venous area	1505 (510-4320)

Table S4. SuPAR level with respect to the survival status (training cohort)

	**SuPAR level Hepatic vein (HV)**	**SuPAR level Portal vein (PV)**
3-month survival		
yes: 88 %	5.08 (1.90-15.96)	4.60 (1.22-13.75)
no: 12 %	14.14 (5.27-17.76)	9.62 (4.85-16.84)
6-month survival		
yes: 76 %	4.73 (1.90-11.91)	4.36 (1.22-13.75)
no: 24 %	8.77 (3.68-17.76)	7.72 (4.04-16.84)
12-month survival		
yes: 61 %	4.64 (1.90-11.28)	3.95 (1.22-13.08)
no: 39 %	7.06 (3.13-17.76)	6.66 (2.16-16.84)
24-month survival		
yes: 37 %	3.44 (1.90-9.16)	3.75 (1.58-13.08)
no: 63 %	6.14 (1.99-17.76)	5.50 (1.22-16.84)

Data are shown as median and ranges

Table S5: Comparison of patient characteristics between patients with high or low suPAR levels (median)

Parameter	Training cohort			Validation cohort		
	**HV suPAR < median (5.27ng/ml)**	**HV suPAR > median (5.27ng/ml)**	**p-value**	**suPAR < median (8.93 ng/ml)**	**suPAR > median (8.93 ng/ml)**	**p-value**
**Age** (years, median, IQR)	59.5 (15)	59.0 (12)	0.706	61 (13)	57 (12)	0.122
**BMI** (kg/m^2, median, IQR)	25.65 (5.23)	24.02 (5.27)	0.354	N/A*	N/A*	-
**Sex** (%)						
male	68.2	66.0		77.3	76.0	
female	31.8	34.0	0.822	22.7	24.0	1.000
**TIPS indication** (%)						
Ascites	27.7	60.9		88.0	81.3	
Variceal bleeding	53.2	26.1		12.0	16.0	
Variceal bleeding and ascites	8.5	10.8		0.0	2.7	
HRS	10.6	2.2	0.005	0.0	0.0	0.320
**Child-Pugh** (%)						
Class A	25.5	8.7		8.0	4.0	
Class B	70.2	60.7		69.3	60.0	
Class C	4.3	30.4	0.001	22.7	36.0	0.140
**Etiology** (%)						
Alcohol-related	70.7	80.4		73.3	86.7	
NAFLD	-	-		9.3	5.3	
Cholestatic liver disease	2.4	4.3		2.7	2.7	
Viral	9.8	13.0		2.7	0.0	
Other	17.1	2.2	0.182	12.0	5.3	0.250

*As the majority of patients had tense ascites, BMI was not assessed in the validation cohort.

Table S6: Uni- and multivariate Cox-regression analyses for overall survival including the MELD-Na score (training cohort)

	Univariate regression models		Multivariate regression model	
**Parameter**	**p-value**	**Hazard ratio (CI)**	**p-value**	**Hazard ratio (CI)**
suPAR HVs	<0.001	1.299 (1.198-1.409)	0.001	1.215 (1.085-1.359)
MELD-Na	<0.001	1.112 (1.069-1.156)	0.029	1.057 (1.006-1.111)
Potassium	0.034	1.434 (1.028-2.000)	0.049	1.404 (1.002-1.968)

Table S7: Uni- and multivariate Cox-regression analyses for overall survival including the FIPS score (training cohort)

	Univariate regression models		Multivariate regression model	
**Parameter**	**p-value**	**Hazard ratio (CI)**	**p-value**	**Hazard ratio (CI)**
suPAR HV	<0.001	1.299 (1.198-1.409)	<0.001	1.247 (1.127-1.380)
Sodium	0.004	0.927 (0.881-0.976)	0.408	0.977 (0.923-1.033)
Potassium	0.034	1.434 (1.028-2.000)	0.159	1.282 (0.907-1.811)
FIPS score	<0.001	1.888 (1.363-2.617)	0.073	1.401 (0.969-2.027)

FIPS: Freiburg index of post-TIPS survival

Table S8: Uni- and multivariate Cox-regression analyses for transplant-free survival (training cohort)

	Univariate regression models		Multivariate regression model	
**Parameter**	**p-value**	**Hazard ratio (CI)**	**p-value**	**Hazard ratio (CI)**
suPAR HV	<0.001	1.287 (1.192-1.390)	0.001	1.190 (1.075-1.317)
suPAR PV	<0.001	1.173 (1.097-1.255)		
Creatinine	<0.001	1.660 (1.361-2.025)	0.096	1.251 (0.961-1.628)
INR	0.191	1.686 (0.771-3.688)		
Bilirubin	0.003	1.266 (1.081-1.481)	0.048	1.198 (1.002-1.432)
ALT	0.882	1.001 (0.988-1.014)		
AST	0.370	0.992 (0.973-1.010)		
GGT	0.180	0.999 (0.997-1.001)		
Albumin	0.809	0.996 (0.964-1.029)		
Sodium	0.001	0.919 (0.875-0.965)	0.100	0.959 (0.912-1.008)
Potassium	0.033	1.415 (1.028-1.947)	0.044	1.440 (1.009-2.055)
Leucocyte count	0.049	1.092 (1.000-1.191)	0.876	1.008 (0.911-1.116)
Thrombocyte count	0.511	1.001 (0.998-1.005)		
Age	0.117	1.025 (0.995-1.050)		
BMI	0.752	1.013 (0.935-1.097)		
Sex	0.978	1.007 (0.632-1.628)		
