# Supplementary material for: Soluble urokinase plasminogen activator receptor levels predict survival in patients with portal hypertension undergoing TIPS
Source: JHEP Rep. 2024 Mar 4;6(5):101054. doi: 10.1016/j.jhepr.2024.101054 (PMC11053213; doi:10.1016/j.jhepr.2024.101054)
Supplement: Multimedia component 2 [file mmc2.docx]

**JHEP Reports**

**CTAT methods**

Tables for a “Complete, Transparent, Accurate and Timely account” (CTAT) are now mandatory for all revised submissions. The aim is to enhance the reproducibility of methods.

- Only include the parts relevant to your study
- Refer to the CTAT in the main text as ‘Supplementary CTAT Table’
- Do not add subheadings
- Add as many rows as needed to include all information
- Only include one item per row

**If the CTAT form is not relevant to your study, please outline the reasons why:**

|  |
| --- |

- 1. **Antibodies**

| **Name** | **Citation** | **Supplier** | **Cat no.** | **Clone no.** |
| --- | --- | --- | --- | --- |
| **None** |  |  |  |  |

- 1. **Cell lines**

| **Name** | **Citation** | **Supplier** | **Cat no.** | **Passage no.** | **Authentication test method** |
| --- | --- | --- | --- | --- | --- |
| **None** |  |  |  |  |  |

- 1. **Organisms**

| **Name** | **Citation** | **Supplier** | **Strain** | **Sex** | **Age** | **Overall n number** |
| --- | --- | --- | --- | --- | --- | --- |
| **None** |  |  |  |  |  |  |

- 1. **Sequence based reagents**

| **Name** | **Sequence** | **Supplier** |
| --- | --- | --- |
| **None** |  |  |

- 1. **Biological samples**

| **Description** | **Source** | **Identifier** |
| --- | --- | --- |
| **Serum / blood samples of patients receiving TIPS (including controls) as specified in materials and methods (page 8-10)** | **Human** | **N/A** |

- 1. **Deposited data**

| **Name of repository** | **Identifier** | **Link** |
| --- | --- | --- |
| **None** |  |  |

- 1. **Software**

| **Software name** | **Manufacturer** | **Version** |
| --- | --- | --- |
| SPSS | (SPSS Inc., Chicago, IL, USA) | Versions 22 and 29 |
| GraphPad Prism | GraphPad Software, San Diego, CA, USA | 7.0 and 8.0 |

- 1. **Other (*e.g*. drugs, proteins, vectors etc.)**

| ELISA for suPAR | suPARnostic, ViroGates, Birkerød, Denmark | Nr. A001 |
| --- | --- | --- |

- 1. **Please provide the details of the corresponding methods author for the manuscript:**

| Prof. Dr. Christoph Roderburg  Clinic for Gastroenterology, Hepatology and Infectious Diseases,  University Hospital Düsseldorf,  40225 Düsseldorf,  Germany  [Christoph.roderburg@med.uni-duesseldorf.de](mailto:Christoph.roderburg@med.uni-duesseldorf.de)  +49-211-81-16330 |
| --- |

**2.0 Please confirm for randomised controlled trials all versions of the clinical protocol are included in the submission. These will be published online as supplementary information.**

| **N/A** |
| --- |
