# Supplementary material for: Soluble urokinase plasminogen activator receptor levels predict survival in patients with portal hypertension undergoing TIPS
Source: JHEP Rep. 2024 Mar 4;6(5):101054. doi: 10.1016/j.jhepr.2024.101054 (PMC11053213; doi:10.1016/j.jhepr.2024.101054)
Supplement: Multimedia component 4 [file mmc4.pdf]

Soluble urokinase plasminogen activator receptor levels predict survival in patients with portal hypertension undergoing TIPS

Sven H. Loosen,[1,†] **Fabian Benz**,[2,†] **Raphael Mohr**,[2,5,†] **Philipp A. Reuken**,[3,†] **Theresa H. Wirtz**,[4,†] Lioba Junker,[4] Christian Jansen,[5] Carsten Meyer,[6] Michael Praktiknjo,[7] Alexander Wree,[2] Johanna Reißing,[4] Münevver Demir,[2] Wenyi Gu,[7] Mihael Vucur,[1] Robert Schierwagen,[7] Andreas Stallmach,[3] Anselm Kunstein,[1] Johannes Bode,[1] Christian Trautwein,[4] Frank Tacke,[2] Tom Luedde,[1] Tony Bruns,[4,‡] Jonel Trebicka,[7,8,9,‡] Christoph Roderburg[1,2,*,‡]

[1]Department of Gastroenterology, Hepatology and Infectious Diseases, University Hospital Düsseldorf, Medical Faculty of Heinrich Heine University Düsseldorf, 40225 Düsseldorf, Germany; [2]Department of Gastroenterology and Hepatology, Campus Virchow Klinikum and Campus Charité Mitte, Charité Universitätsmedizin Berlin, 13353 Berlin, Germany; [3]Department of Internal Medicine IV, Jena University Hospital, Am Klinikum 1, 07747 Jena, Germany; [4]Department of Medicine III, University Hospital RWTH Aachen, Pauwelsstrasse 30, 52074 Aachen, Germany; [5]Department of Internal Medicine I, University Clinic Bonn, Bonn, Germany; [6]Department of Radiology, University Clinic Bonn, Bonn, Germany; [7]Department of Internal Medicine B, University of Münster, Münster, Germany; [8]European Foundation for the Study of Chronic Liver Failure - EF CLIF, Barcelona, Spain; [9]Department of Gastroenterology and Hepatology, Odense University Hospital, Odense, Denmark

JHEP Reports **2024.** https://doi.org/10.1016/j.jhepr.2024.101054

Background & Aims: Transjugular intrahepatic portosystemic shunt (TIPS) is the most effective therapy for complications of portal hypertension. However, clinical outcomes following TIPS placement vary widely between patients and identifying ideal candidates remains a challenge. Soluble urokinase plasminogen activator receptor (suPAR) is a circulating marker of immune activation that has previously been associated with liver inflammation, but its prognostic value in patients receiving TIPS is unknown. In the present study, we evaluated the potential clinical relevance of suPAR levels in patients undergoing TIPS insertion.
Methods: suPAR concentrations were measured by ELISA in hepatic vein (HV) and portal vein (PV) blood samples from 99 patients (training cohort) as well as peripheral venous blood samples from an additional 150 patients (validation cohort) undergoing TIPS placement. The association between suPAR levels and patient outcomes was assessed using Kaplan-Meier methods and Cox-regression analyses.
Results: suPAR concentrations were significantly higher in HV samples compared to PV samples and correlated with PV concentration, the presence of ascites, renal injury, and consequently with the Child-Pugh and MELD scores. Patients with lower suPAR levels had significantly better short- and long-term survival after TIPS insertion, which remained robust after adjustment for confounders in multivariate Cox-regression analyses. Sensitivity analysis showed an improvement in risk prediction in patients stratified by Child-Pugh or MELD scores. In an independent validation cohort, higher levels of suPAR predicted poor transplant-free survival after TIPS, particularly in patients with Child-Pugh A/B cirrhosis.
Conclusion: suPAR is largely derived from the injured liver and its levels are predictive of outcome in patients undergoing TIPS. suPAR, as a surrogate of hepatic inflammation, may be used to stratify care in patients following TIPS insertion.
Impact and implications: Transjugular intrahepatic portosystemic shunt (TIPS) is the most effective therapy for complications of portal hypertension. However, clinical outcomes following TIPS placement vary widely between patients and identification of the ideal candidates remains challenging. We show that soluble urokinase plasminogen activator receptor (suPAR), a circulating marker of immune activation that can easily be measured in routine clinical practice, is a novel marker to identify patients who will benefit from TIPS and those who will not.
© 2024 The Author(s). Published by Elsevier B.V. on behalf of European Association for the Study of the Liver (EASL). This is an open access article under the CC BY license (http://creativecommons.org/licenses/by/4.0/).

Introduction

Portal hypertension is a well-known cause of complications and decompensation in patients with cirrhosis. Transjugular intrahepatic portosystemic shunt (TIPS) is the most effective measure to reduce portal pressure and treat complications of portal hypertension, resulting in a survival benefit in well-selected patients.[1–3] However, portal hypertension is not the only driver of complications; increasing and exaggerated systemic inflammation is also associated with decompensation and particularly acute-on-chronic liver failure (ACLF).[4,5] Even when portal hypertension

Keywords: suPAR; TIPS; portal hypertension; survival; biomarker.
Received 29 April 2023; received in revised form 10 December 2023; accepted 12 January 2024; available online 4 March 2024
† These authors share first authorship
‡ These authors share senior authorship
* Corresponding author. Address: Clinic for Gastroenterology, Hepatology and Infectious Diseases, University Hospital Düsseldorf, 40225 Düsseldorf, Germany; Tel.: +49-211-81-16330.
E-mail address: Christoph.roderburg@med.uni-duesseldorf.de (C. Roderburg).

after TIPS is adequately treated, hepatic-derived inflammation appears to be the driver of organ failure and decompensation in patients with cirrhosis.[6] Several approaches have been proposed to assess hepatic and systemic inflammation, but single markers do not seem to predict outcome in acute decompensation.[7]

The soluble urokinase plasminogen activator receptor (suPAR) is a part of the urokinase plasminogen activator/urokinase plasminogen activator receptor signaling cascade.[8] It is frequently shed from activated innate immune cells under inflammatory conditions and regulates various immune signaling cascades such as cellular differentiation, migration, adhesion and invasion.[9] Several studies have demonstrated the diagnostic and prognostic relevance of elevated suPAR levels in inflammatory and cardiovascular diseases as well as cancer.[10–12] Elevated suPAR levels have been associated with hepatic inflammation and fibrosis in patients with cirrhosis.[13] In patients with decompensated cirrhosis, suPAR levels correlate with organ failure and inflammation and are associated with poor short-term survival.[14,15] Moreover, intrahepatic suPAR activation and circulating suPAR levels have been suggested as biomarkers in patients with acute liver failure.[16] In contrast, the potential relevance of circulating suPAR concentrations in patients undergoing TIPS implantation is poorly understood.

Therefore, in the present study, we assessed suPAR levels in portal venous, hepatic venous and systemic blood in two different cohorts of patients undergoing TIPS placement to elucidate a potential role of suPAR as a novel tool to stratify eligible candidates pre-intervention based on their predicted outcomes after TIPS implantation.

Table 1. Patient characteristics of the training and validation cohort.

Cohort	Training cohort	Validation cohort
Number of patients (n)	99	150
Age (years, median and IQR)	59 (53-65)	59 (54-66)
Sex, n (%)		
Female	32 (33.3)	35 (23.3)
Male	64 (66.7)	115 (76.7)
TIPS indication, n (%)		
Recurrent/refractory ascites	43 (43.9)	127 (84.7)
Variceal bleeding	39 (39.8)	21 (14.0)
Variceal bleeding and ascites	9 (9.2)	2 (1.3)
Hepatorenal syndrome	7 (7.1)	–
Etiology, n (%)		
Alcohol-related liver disease	69 (75)	120 (80.0)
Non-alcoholic fatty liver disease	–	11 (7.3)
Cholestatic liver disease	3 (3.3)	4 (2.7)
Viral	11 (12)	2 (1.3)
Other	9 (9.7)	13 (8.7)
Child-Pugh, n (%)		
Class A	17 (17.3)	9 (6.0)
Class B	65 (66.3)	97 (64.7)
Class C	16 (16.3)	44 (29.3)
Transplant-free survival		
At 6 months	73.8 (4.7)	77.1 (3.7)
At 12 months	58.8 (5.3)	70.1 (4.4)
At 24 months	34.6 (5.1)	58.8 (5.4)
At 48 months	22.6 (4.5)	53.3 (6.3)
Overall survival		
At 6 months	75.8 (4.6)	83.5 (3.4)
At 12 months	61.0 (5.3)	78.4 (4.0)
At 24 months	37.4 (5.4)	68.8 (5.4)
At 48 months	25.3 (4.9)	62.1 (6.7)

Baseline characteristics are depicted as frequencies or median (IQR). Transplant-free and overall survival is depicted as Kaplan-Meier estimates with standard error. Analysis of overall survival was right-censored at liver transplantation. TIPS, transjugular intrahepatic portosystemic shunt.

Materials and methods

Study design

A total of 99 patients with cirrhosis and severe portal hypertension scheduled for TIPS insertion were enrolled at the Department of Internal Medicine I at University Clinic Bonn (Germany) between 1996 and 2003.[15,16] Clinical characteristics are shown in Table 1. Inclusion criteria were: 1. Age between 18 and 80 years; 2. Decompensated cirrhosis with an indication for TIPS. Decompensated cirrhosis is defined as an acute deterioration of liver function in a patient with cirrhosis and is characterized by ascites, hepatic encephalopathy, or variceal bleeding. Exclusion criteria included: Clinically determined contraindications for TIPS placement, such as severe heart failure, severe pulmonary hypertension, active systemic infection, spontaneous bacterial peritonitis, overt hepatic encephalopathy or other medical conditions that would render the procedure technically impossible. One to three weeks after TIPS insertion, an invasive control of the TIPS was performed as part of routine care. The median follow-up was 442 days (IQR 164-1071). The study protocol was approved by the local ethics committee of the University of Bonn (029/13) and was conducted in accordance with the Declaration of Helsinki. Written informed consent was obtained from all patients.

TIPS procedure and hemodynamic measurements

TIPS (8-10 mm Wallstent, Boston Scientific, Massachusetts, USA) placement was performed as previously described in detail.[15–17] A single injection of antibiotic prophylaxis (cefuroxime 1.5 g) was administered during the TIPS procedure. Portal and hepatic venous pressures were measured invasively using a pressure transducer system (Combitrans, Braun Melsung, Germany) and a multichannel monitor (Sirecust, Siemens, Germany). The difference between portal and hepatic venous pressure was defined as the hepatic venous pressure gradient (HVPG). Arterial pressure and heart rate were monitored non-invasively. Laboratory parameters as well as portal and systemic hemodynamics were recorded (Tables S1 and S2). Blood samples were collected from the portal vein (PV) and hepatic vein (HV) as previously described in detail.[15–17] Hepatic venous samples were collected from the respective HV selected for TIPS placement prior to the puncture of the PV. Blood samples from the PV were collected immediately after PV puncture, prior to tract dilation or TIPS insertion. Immediately after collecting whole blood from the PV and HV, samples were centrifuged at 2000 g for 10 min. Serum samples were then stored at −80 °C until further use.

External validation cohort

We retrospectively analyzed cubital vein serum from an independent cohort of 150 patients who underwent TIPS insertion using ePTFE-covered VIATORR® stents at the Jena University Hospital between October 2013 and September 2022 or at the University Hospital RWTH Aachen between August 2019 and May 2023 (Table 1). Serum samples were allowed to clot for 30 min at room temperature, centrifuged at 1000 × g for 10 min, and stored at −80 °C until further analysis. The median time between blood collection and TIPS insertion was 0 days (IQR 0 to 1). In addition, peripheral venous blood was collected in a subset of patients the morning after TIPS insertion. Patients were followed until death or liver transplantation. Written informed consent was obtained from patients prior to enrolment. The

study conformed to the ethical guidelines of the 1975 Declaration of Helsinki and was approved by the internal review board (Ethics committee of the Jena University Hospital, no. 3683-02/3, 2019-1510, 2018-1080-BO) and the University Hospital RWTH Aachen (no. EK023-19).

Measurements of circulating suPAR levels

Concentrations of suPAR in serum samples from the PV, HV, and peripheral vein were determined using a commercially available ELISA following the manufacturer's instructions (Nr. A001, suPARnostic, ViroGates, Birkerød, Denmark).

Statistical analysis

All statistical analyses were performed using SPSS 22 and 29 (SPSS Inc., Chicago, IL, USA) and visualized using GraphPad Prism 7.0 and 8.0 (GraphPad Software, San Diego, CA, USA). Data are presented as mean ± SEM or median and range. We used the non-parametric Wilcoxon signed-rank test to compare paired data, the Mann-Whitney *U* test for unpaired comparisons of two groups and the Kruskal–Wallis ANOVA and Mann-Whitney *U* test for *post hoc* analysis for comparison of more than two groups. Box or violin plots illustrate comparisons between subgroups, displaying a statistical summary of median, quartiles and extreme values. Whiskers are drawn up to the largest observed point from the dataset with a distance of 1.5x the IQR. Correlations were analyzed using the Spearman correlation coefficient. The prognostic value of the variables was tested by uni- and multivariable analyses using a Cox proportional hazards regression model (forward stepwise likelihood-quotient). Survival rates are shown using Kaplan-Meier plots and analyzed by log-rank test. For analysis of transplant-free survival in the validation cohort, data were right-censored at loss-to-follow-up or at 5 years. Receiver-operating characteristic (ROC) curve analyses and the derived AUC statistic were generated by plotting sensitivity against 1 – specificity. *P* values <0.05 in two-sided tests were considered statistically significant (*$p < 0.05$; **$p < 0.01$; ***$p < 0.001$).

Results

Patient characteristics

A total of 99 patients with decompensated cirrhosis were included into the training cohort. The median age was 59 years (IQR 53-65 years); 66.7% of patients were male. Alcohol-related liver disease was the most common etiology of cirrhosis (75.0%). 17% of patients presented with Child-Pugh stage A, 66% with stage B and 16% with stage C cirrhosis. The median model for end-stage liver disease (MELD) score was 10 (range: 6-33). Most patients had esophageal varices (I-II°: 67%; III-IV°: 22%) as well as ascites (mild: 17%, moderate to severe: 64%). Hepatorenal syndrome (HRS) was observed in 23% of patients and 15% of patients had experienced at least one episode of hepatic encephalopathy (HE). Indications for TIPS implantation were refractory ascites (43.9%), variceal bleeding (39.8%), variceal bleeding and refractory ascites (9.2%), and HRS (7.1%). For the external validation cohort, we enrolled 150 patients with decompensated cirrhosis. Median age was 59 years (IQR 54-66 years); 76.7 were male; 64.7% and 29.3% had Child-Pugh B and C cirrhosis, respectively. Alcohol-related liver disease (80.0%) was the most prevalent etiology of cirrhosis. Table 1 and Tables S1-4 provide a detailed summary of demographic, clinical, laboratory and hemodynamic patient characteristics.

Hepatic vein suPAR levels are elevated in patients with cirrhosis and correlate with portal vein concentrations

Based on existing data showing a significant elevation of circulating suPAR levels in patients with chronic liver disease,[18] we first analyzed suPAR concentrations in HV and PV blood samples of patients with cirrhosis and portal hypertension scheduled for TIPS insertion. Interestingly, in these patients, suPAR concentrations were significantly higher in the HV compared to the PV, suggesting a predominantly hepatic origin of this inflammatory marker (Fig. 1A). suPAR concentrations in HV and PV blood showed a significant positive correlation ($r_S = 0.83$, $p < 0.001$, Fig. 1B). Interestingly, we observed a gradual increase in HV suPAR concentrations with increasing stage of cirrhosis from 2.92 ng/ml in patients with Child-Pugh A cirrhosis to 7.34 ng/ml in those with Child-Pugh C cirrhosis (Fig. 1C). In line, HV suPAR concentrations increased stepwise in patients with a higher MELD category (Fig. 1D) and showed a strong positive correlation with the MELD score (Fig. 1E). A similar correlation in terms of circulating suPAR concentrations and severity of liver disease was observed for PV blood samples (Fig. S1A-C). A comparison of

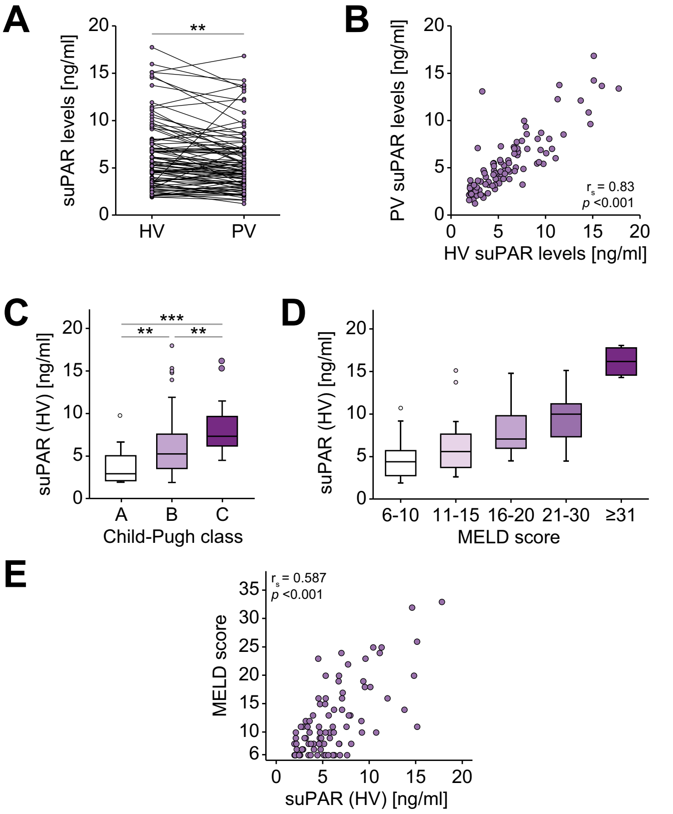

Fig. 1. Hepatic and portal vein suPAR concentrations in patients with chronic liver disease. (A) suPAR concentrations are significantly higher in HV compared to PV samples (Wilcoxon signed-rank test). (B) HV and PV suPAR levels significantly correlate in patients with chronic liver disease (Spearman correlation coefficient). (C) HV suPAR concentrations show a significant, stepwise increase in patients with a more advanced Child-Pugh class (Kruskal–Wallis ANOVA, post hoc: Mann-Whitney U-test) . (D) HV suPAR concentrations show a stepwise increase in patients with a more advanced MELD score. (E) HV suPAR concentrations correlate with the patients' MELD score (Spearman correlation coefficient). HV, hepatic vein; MELD, model of end-stage liver disease; PV, portal vein; suPAR, soluble urokinase plasminogen activator receptor; ** p <0.01; *** p <0.001.

patient characteristics with high or low HV suPAR levels stratified by the median HV suPAR level of the cohort (5.27 ng/ml) is shown in Table S5.

To determine possible causes of elevated suPAR levels in patients with cirrhosis, we next compared HV and PV suPAR levels in different subgroups of patients. While suPAR levels were comparable in male and female patients, in patients younger or older than 65 years, and in patients with or without alcohol-related liver disease (Fig. S2A-C and Fig. 3A-C), we observed significantly higher HV and PV suPAR levels in patients with moderate to severe ascites compared to patients without or with mild ascites (Fig. S2D and Fig. 3D). SuPAR levels were also significantly higher in patients with HRS compared to patients with normal renal function (Fig. S2E and Fig. 3E). Patients with or without esophageal varices or hepatic encephalopathy had comparable HV and PV suPAR concentrations (Fig. S2F,G and Fig. 3F,G). We finally performed extensive correlation analyses between HV and PV suPAR concentrations and various laboratory and hemodynamic parameters (Table 2). Both HV and PV suPAR levels positively correlated with systemic creatinine and urea, suggesting impaired renal function as another driver of elevated suPAR levels. In addition, there was a positive correlation between suPAR and bilirubin, cholinesterase, ammonia concentrations as well as the leucocyte count, while sodium levels negatively correlated with HV and PV suPAR levels (Table 2). In addition, HV and PV suPAR levels positively correlated with coagulation parameters such as the partial thromboplastin time and international normalized ratio (HV suPAR only) as well as scoring systems of liver function (MELD and Child-Pugh scores, Fig. 2). In contrast, suPAR concentrations did not correlate with potassium, aspartate aminotransferase, alanine aminotransferase, gamma-glutamyltransferase, albumin or thrombocyte levels. Moreover, HV and PV suPAR concentrations did not correlate with hemodynamic parameters such as portal pressure, HVPG or portal venous velocity.

Hepatic and portal vein suPAR levels predict outcome in patients with cirrhosis undergoing TIPS

We subsequently hypothesized that circulating suPAR concentrations might serve as a novel prognostic marker in patients undergoing TIPS. We therefore compared pre-interventional suPAR levels of patients who survived or died within the first 3, 6, 12, or 24 months after TIPS insertion. In our cohort, 3-, 6-, 12- and 24-month survival rates were 88%, 76%, 61% and 37%, respectively (Table S4). At all four time points, HV and PV suPAR concentrations were significantly higher in patients who died during each period compared to those who were still alive (Fig. 2A-D and Fig. S4A-D). ROC curve analyses revealed that both HV and PV suPAR levels had a higher or comparable prognostic relevance for predicting patient survival compared to the MELD-Na score (Fig. 2 E-H). Interestingly, the combinational use of HV suPAR levels and the MELD-Na score showed a higher AUC value compared to either parameter alone (Fig. 2E,F). In the next step, we used Kaplan-Meier curve analyses to investigate the prognostic significance of HV and PV suPAR concentrations in terms of overall survival (OS) after TIPS insertion. We subdivided our cohort of patients according to the median as well as lower/upper quartile of suPAR levels. Importantly, the median OS was significantly reduced in patients with HV suPAR concentrations above the median (Fig. 2I) as well as above the lower/upper quartile (Fig. 2J,K). Comparable results were observed for PV suPAR concentrations (Fig. S4E-G).

To exclude potential confounders on the prognostic role of suPAR levels, we performed uni- and multivariable Cox-regression regression models. We included a variety of potentially prognostic factors into univariate analyses to predict overall survival in patients with chronic liver disease after the TIPS procedure. Here, both HV (hazard ratio [HR] 1.299, 95% CI 1.198-1.409, p < 0.001) and PV suPAR levels (HR 1.168, 95% CI 1.089-1.252, p < 0.001) were a significant predictor for OS (Table 3). Other significant predictors for OS included serum creatinine, bilirubin, sodium and potassium levels (Table 3). Subsequently, we included these parameters together with HV suPAR levels into a multivariate Cox-regression model. This analysis revealed that HV suPAR levels represent an independent predictor of OS following TIPS insertion (HR 1.235, 95% CI 1.100-1.387, p < 0.001, Table 3). In a second multivariable model that included the MELD-Na score instead of its components, the prognostic relevance of HV suPAR levels was confirmed (HR 1.215, 95% CI 1.085-1.359, p = 0.001, Table S6). In addition, a third multivariate model revealed that HV suPAR levels were independent of the FIPS (Freiburg index of post-TIPS survival) score (HR 1.247, 95% CI 1.127-1.380, p < 0.001, Table S7). Importantly, HV suPAR levels were not only a strong predictor for OS but also for transplant-free survival in a multivariate Cox-regression model (HR 1.190, 95% CI 1.075-1.317, p = 0.001, Table S8).

We finally hypothesized that the individual ratio between HV and PV suPAR concentrations might also be of prognostic relevance and compared the numerical difference of HV and PV (delta suPAR = HV-PV) between patients who did or did not survive the 3-, 6-, 12-, or 24-month period following TIPS placement. However, no significant alterations of delta suPAR became apparent (Fig. S5A-D). In line, Kaplan-Meier curve analysis did not reveal a survival benefit for patients with either a positive or negative delta suPAR (Fig. S5E).

SuPAR identifies a subgroup of patients with advanced cirrhosis who experience poor outcomes after TIPS insertion

The Child-Pugh score is a clinically established tool for predicting prognosis in patients with cirrhosis. Finally, in an exploratory analysis, we investigated whether the prognostic potential of HV and PV suPAR levels could further increase its prognostic relevance. The additional stratification of patients with Child-Pugh A cirrhosis, who are meant to have a comparatively good

Table 2. Correlation analyses between suPAR levels and various laboratory markers before TIPS (training cohort).

	suPAR (hepatic vein)		suPAR (portal vein)	
**Parameter**	r_S	***p* value**	r_S	***p* value**
Sodium	-0.316	0.002	-0.222	0.029
Creatinine	0.534	<0.001	0.589	<0.001
Urea	0.479	<0.001	0.558	<0.001
Bilirubin	0.262	0.012	0.279	0.006
Cholinesterase	-0.637	<0.001	-0.556	<0.001
Leukocyte count	0.289	0.005	0.270	0.007
Ammonia	0.342	0.055	0.365	0.037
PTT	0.267	0.009	0.269	0.008
INR	0.251	0.018	0.195	0.066
MELD score	0.587	<0.001	0.638	<0.001
Child-Pugh score	0.451	<0.001	0.387	<0.001

All correlation analyses were performed using the Spearman correlation coefficient. INR, international normalized ratio; MELD, model of end-stage liver disease; PTT, partial thromboplastin time; suPAR, soluble urokinase plasminogen activator receptor; TIPS, transjugular intrahepatic portosystemic shunt.

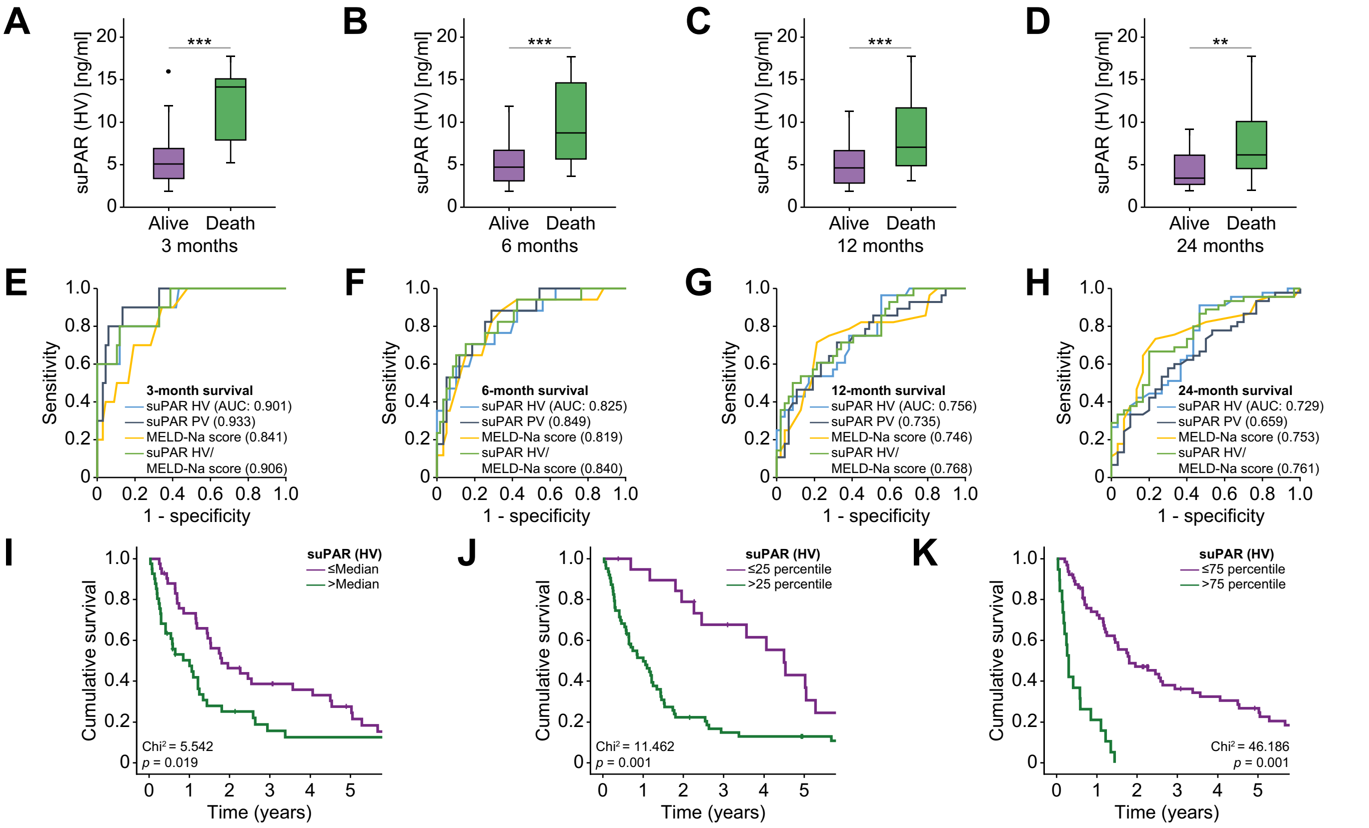

Fig. 2. Hepatic vein suPAR levels correlate with short- and long-term outcomes after TIPS insertion. HV suPAR levels are significantly higher in patients who died within 3 (A), 6 (B), 12 (C), or 24 months (D) after TIPS placement (all Mann-Whitney U-test). (E to H) ROC curve analyses show that both HV and PV suPAR levels have a numerically higher or comparable AUC for predicting patient survival at 3, 6, 12, and 24 months, when compared to the MELD or MELD-Na score. The median overall survival is significantly reduced in TIPS patients with baseline HV suPAR concentrations above the median (I, log-rank-test) or the lower/upper quartile (J, K, both log-rank-test). HV, hepatic vein; MELD, model of end-stage liver disease; PV, portal vein; ROC, receiver-operating characteristic; suPAR, soluble urokinase plasminogen activator receptor; TIPS, transjugular intrahepatic portosystemic shunt.

Table 3. Uni- and multivariate Cox-regression analyses for overall survival (training cohort).

	Univariate regression models		Multivariate regression model	
**Parameter**	***p* value**	**Hazard ratio (CI)**	***p* value**	**Hazard ratio (CI)**
suPAR HV	<0.001	1.299 (1.198-1.409)	<0.001	1.224 (1.101-1.360)
suPAR PV	<0.001	1.168 (1.089-1.252)		
Creatinine	<0.001	1.580 (1.306-1.912)	0.267	1.153 (0.897-1.482)
INR	0.133	1.942 (0.817-4.616)		
Bilirubin	0.001	1.302 (1.107-1.532)	0.017	1.264 (1.044-1.532)
ALT	0.681	1.003 (0.990-1.016)		
AST	0.516	0.994 (0.975-1.013)		
GGT	0.247	0.999 (0.997-1.001)		
Albumin	0.941	0.999 (0.965-1.034)		
Sodium	0.004	0.927 (0.881-0.976)	0.535	0.984 (0.935-1.036)
Potassium	0.034	1.434 (1.028-2.000)	0.026	1.473 (1.048-2.071)
Leucocyte count	0.098	1.080 (0.986-1.183)		
Thrombocyte count	0.448	1.001 (0.998-1.005)		
Age	0.067	1.027 (0.998-1.057)		
BMI	0.947	1.003 (0.921-1.093)		
Sex	0.751	0.921 (0.553-1.533)		
Portal/hepatic pressure gradient pre-TIPS	0.588	1.015 (0.961-1.072)		
Portal hepatic pressure gradient post-TIPS	0.408	1.029 (0.962-1.100)		
Portal pressure pre-TIPS	0.055	1.038 (0.999-1.078)		
Portal pressure post-TIPS	0.140	1.032 (0.990-1.075)		
Portal venous velocity pre-TIPS	0.558	1.013 (0.970-1.057)		
Portal venous velocity post-TIPS	0.289	1.010 (0.992-1.029)		

ALT, alanine aminotransferase; AST, aspartate aminotransferase; GGT, gamma-glutamyltransferase; HV, hepatic vein; INR, international normalized ratio; suPAR, soluble urokinase plasminogen activator receptor.

prognosis, according to the individual HV suPAR concentration identified a subgroup of patients ("*Child-Pugh A, HV suPAR high*") with a significantly impaired post-interventional median OS (Fig. 3A). Interestingly, this Child-Pugh A subgroup showed a lower median OS compared to patients with Child-Pugh B or even Child-Pugh C cirrhosis (Fig. 3A-C). Similar results were observed for Child-Pugh B, where HV suPAR levels could also significantly discriminate between a subgroup with a good or poor prognosis (Fig. 3B). This finding was not observed in patients with Child-Pugh C cirrhosis (Fig. 3C). Considering the number of Child-Pugh stage-stratified deaths after 3, 6, 12, and 24 months, the additional prognostic relevance of circulating HV suPAR levels especially for patients with Child-Pugh stage A and B became apparent (Fig. 3D). In line, these findings were confirmed for circulating PV suPAR levels (Fig. S6A-D).

High concentrations of suPAR in peripheral vein blood at TIPS placement indicate poor transplant-free and overall survival

In an independent cohort of 150 patients who underwent insertion of ePTFE-coated TIPS for predominantly refractory or recurrent ascites (Table 1), the median suPAR concentration in sera from peripheral venous samples before TIPS insertion was 8.9 ng/ml (IQR 6.6-11.8). suPAR concentrations correlated with MELD score (rs = 0.334; p < 0.001) and the Child-Pugh score (rs = 0.295; p < 0.001) (Fig. 4A-B). In addition, there was a modest correlation with the white blood cell (WBC) count (rs = 0.187; p = 0.022) and C-reactive protein (CRP: rs = 0.198; p = 0.015). In a subset of 56 samples, paired cubital serum samples immediately before and 1 day after TIPS insertion were analyzed. TIPS insertion did not significantly alter circulating suPAR serum concentrations in the short term (Fig. 2C). During follow-up, 34 (22.7%) patients died and 12 (8.0%) underwent liver transplantation resulting in a cumulative estimate of transplant-free survival of 37.1% (standard error 10.6%) and in a cumulative estimate of OS of 43.5% (standard error 12.2%) at 60 months after TIPS insertion. Analysis of the ROC curve and the Youden index revealed 9.6 ng/ml as the optimal cut-off value to discriminate between transplant-free survivors from patients with liver-related endpoints of liver transplantation or death (Fig. 4D).

The HR for death or transplant in patients with cubital vein suPAR concentrations greater or equal to 9.6 ng/ml was 3.07 (95% CI 1.68-5.60, p <0.001) in univariate analysis, while the HR for death from any cause was 3.27 (95% CI 1.61-6.63, p = 0.001). After adjustment for MELD score and age on multivariable Cox-

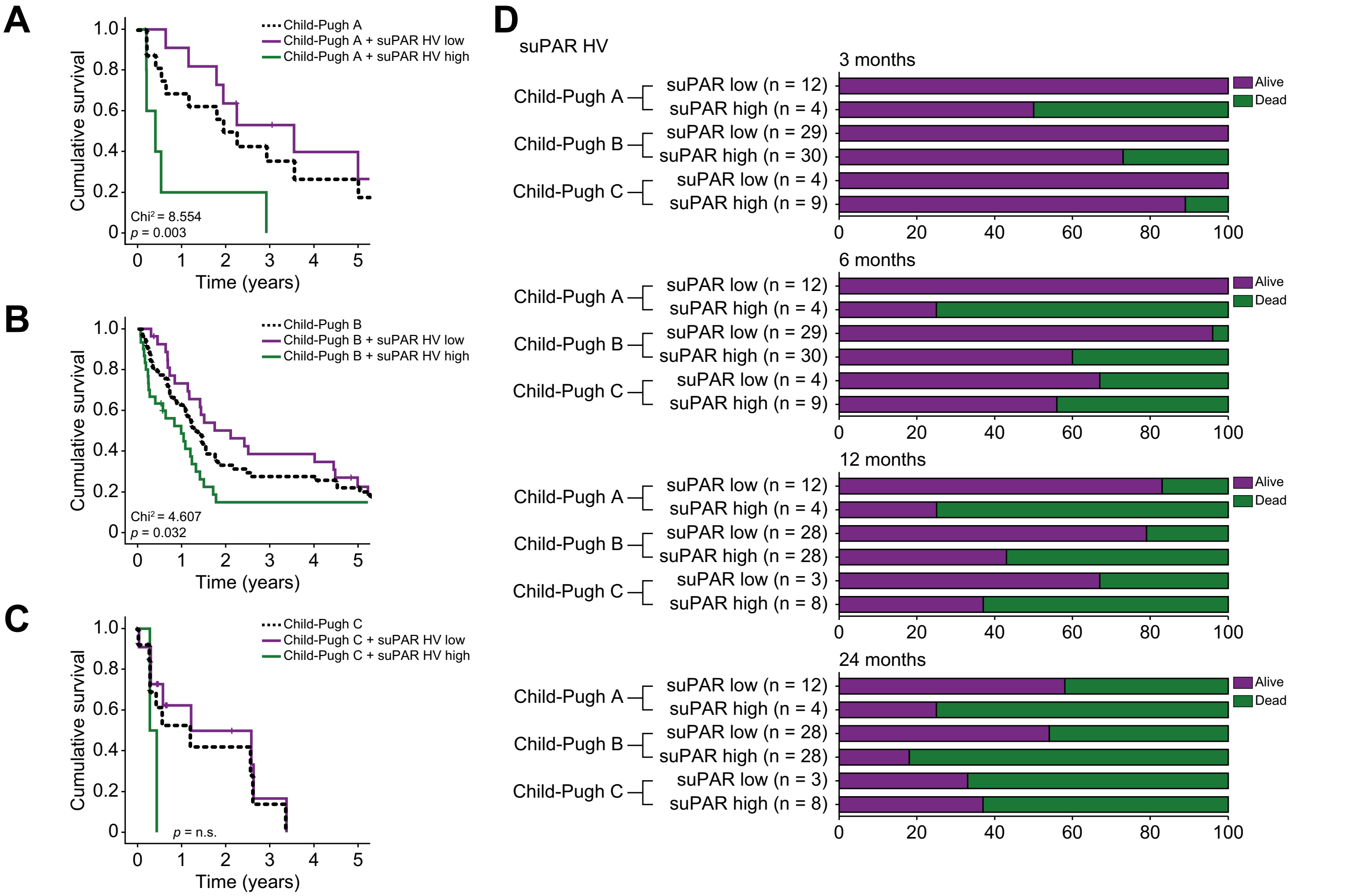

Fig. 3. suPAR identifies a subgroup of patients in early-stage cirrhosis with poor outcomes after TIPS placement. (A) In patients with Child-Pugh A cirrhosis, elevated HV suPAR levels identify a subgroup of TIPS patients with a significantly impaired post-interventional median overall survival, which is lower than in patients with Child-Pugh B and C cirrhosis (log-rank-test). (B) In patients with Child-Pugh B cirrhosis, elevated HV suPAR levels identify a subgroup of TIPS patients with significantly impaired post-interventional outcomes (log-rank-test). (C) This finding is not observed in patients with Child-Pugh C cirrhosis (log-rank-test). (D) Number of Child-Pugh stage-stratified deaths after 3, 6, 12, and 24 months in TIPS patients with high or low baseline HV suPAR levels. HV, hepatic vein; suPAR, soluble urokinase plasminogen activator receptor; TIPS, transjugular intrahepatic portosystemic shunt.

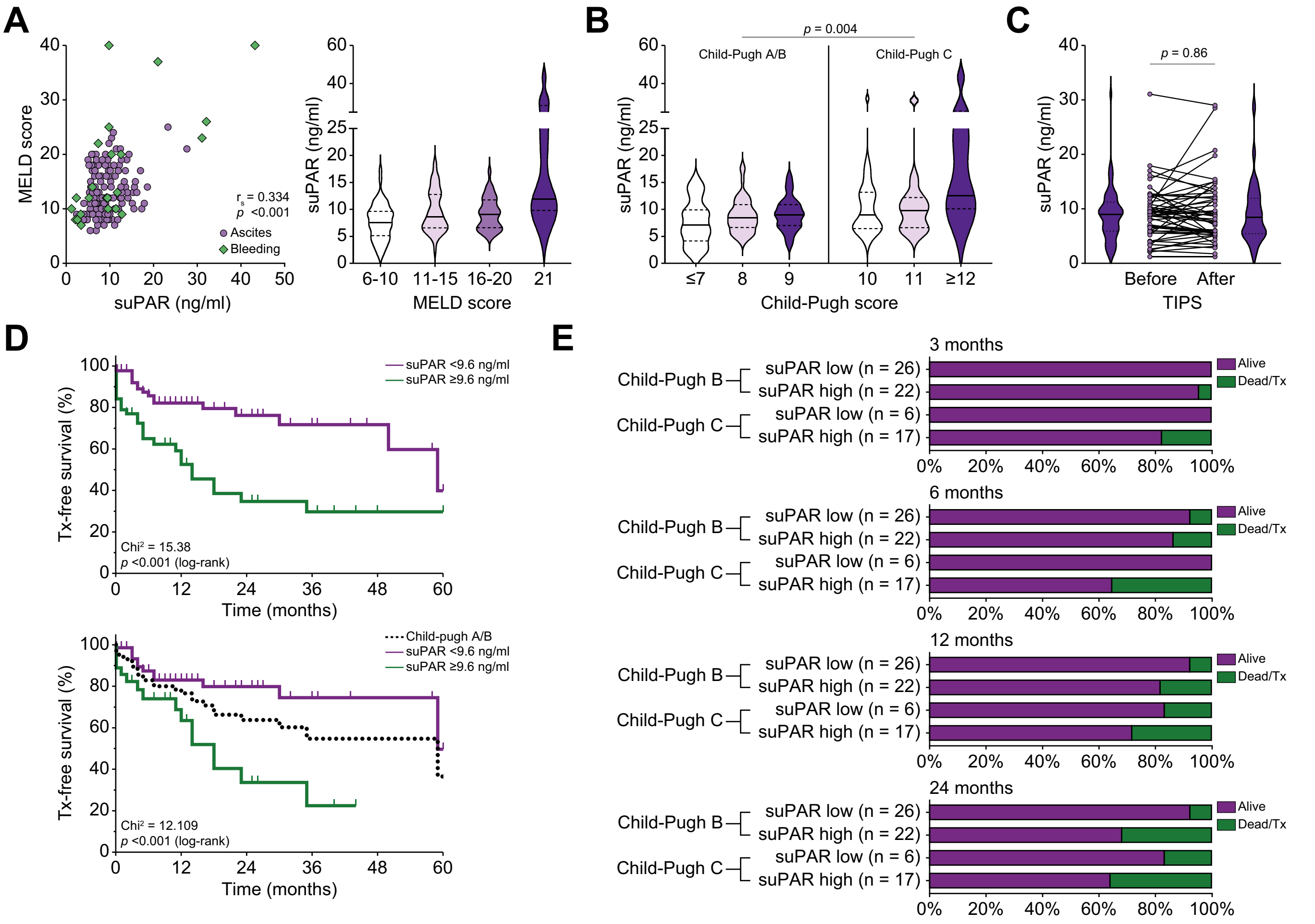

Fig. 4. Correlation of suPAR concentrations with liver function and outcomes after TIPS implantation (validation cohort). (A) Left panel: Scatter plot and correlation analysis of circulating suPAR concentrations, analyzed in sera from peripheral vein blood, with MELD scores in patients receiving ePTFE-covered TIPS insertion for variceal bleeding (black diamonds) or refractory/recurrent ascites (open circles). Spearman's rho with *p* value is indicated. Right panel: Violin plots of circulating suPAR stratified for MELD scores (medians: solid lines, first and third quartiles: dashed lines). (B) Violin plots of suPAR peripheral serum stratified for Child-Pugh scores (medians: solid lines, first and third quartiles: dashed lines). *p* value indicating differences in suPAR levels between patients with Child-Pugh class A/B and C as assessed by Mann-Whitney *U* test. (C) Changes in circulating suPAR concentrations before and 1 day after TIPS insertion. *p* value from Wilcoxon signed-rank test is indicated. (D) Upper panel: Kaplan-Meier analysis of transplant-free survival in 150 patients after TIPS insertion. Death and liver transplantation were considered events, loss to follow-up was right-censored. Patients were stratified for high *vs.* low cubital suPAR concentrations. *p* values from log-rank test. Lower panel: Kaplan-Meier analysis of transplant-free survival in the subgroup of 106 patients with Child-Pugh A or B cirrhosis. *p* values from log-rank test. (E) Number of patients experiencing the events death or transplant 3, 6, 12, and 24 months after TIPS insertion stratified for Child-Pugh class and baseline suPAR concentrations. *For this analysis, patients lost to follow-up were imputed as survivors. MELD, model of end-stage liver disease; suPAR, soluble urokinase plasminogen activator receptor; TIPS, transjugular intrahepatic portosystemic shunt.

regression analysis, the HR for death or transplant in patients with higher cubital vein suPAR concentrations was 2.80 (95% CI 1.51-5.20, p = 0.001), which was similar to the adjusted HR for death from any cause (2.80, 95% CI 1.37-5.75, p = 0.005).

In sensitivity analysis, peripheral vein suPAR concentrations greater or equal to 9.6 ng/ml were very good predictors of transplant-free survival in the subgroup of patients with Child-Pugh class A/B cirrhosis (Chi square 12.11; p <0.001 in log-rank-test) (Fig. 4D) but failed to reach significance in patients with Child-Pugh class C cirrhosis (Chi square 2.18; p = 0.140 in log-rank-test). Patients with Child-Pugh A/B cirrhosis and high suPAR levels had a transplant-free mortality comparable to patients with Child-Pugh C cirrhosis at 12 and 24 months (Fig. 4E).

In a subgroup of 72 patients who received elective TIPS placement for ascites with a Child-Pugh score of 9 or less, MELD score of 15 or less, and total serum bilirubin <3 mg/dl, 24 (33.3%) had cubital suPAR concentrations of 9.6 ng/ml of higher. In this subgroup of patients considered at low risk of mortality after TIPS, higher suPAR also indicated hazard for death or liver transplant after TIPS placement (HR 4.62, 95% CI 1.67-12.79, p = 0.003).

Discussion

This study demonstrates that in patients with complications of portal hypertension, suPAR is predominantly derived from the injured liver, and its levels predict outcome in patients whose

portal hypertension has been adequately treated with TIPS. TIPS implantation is an effective intervention for the treatment of complications of portal hypertension. Although technical advances over the past 30 years have reduced immediate procedural complications, careful patient selection remains key to balancing urgency and efficacy against potential contraindications.[17]

While portal hypertension is the main driver of complications in the compensated stage of advanced chronic liver disease, systemic inflammation is a major driver of complications in the decompensated stage.[18,19] Inflammatory parameters, such as WBC count, acute phase proteins, circulating cytokines, and macrophage markers, have been suggested to improve scoring systems for risk prediction in patients with decompensated cirrhosis and ACLF.[14,19–22]

We have previously shown that circulating suPAR concentrations are a result of cellular activation of neutrophils, monocytes, and macrophages, and correlate with liver function (bilirubin, international normalized ratio, albumin), renal function (creatinine, sodium), and inflammation (WBC count, IL-10, CRP) in patients with decompensated cirrhosis and ascites.[14] Here, we show that suPAR concentrations in PV, HV or cubital blood are indicative of impaired transplant-free and overall survival. Interestingly, the prognostic advantage of suPAR for risk stratification after TIPS was most pronounced in patients with Child-Pugh A/B cirrhosis and less so for Child-Pugh C, indicating the potential prognostic role of immune activation in patients who appear well suited for TIPS according to classical parameters. Notably, we describe a novel subgroup of patients with impaired liver function (Child-Pugh B) but low suPAR levels who have an overall prognosis similar to that of patients with Child-Pugh A cirrhosis. Thus, measurements of suPAR levels allow for a more precise stratification of patients than previous approaches.

Predictors of mortality after TIPS placement are often good predictors of outcome in patients with advanced chronic liver disease and portal hypertension, even in the absence of TIPS placement. Recently, it has been speculated that TIPS may even lead to higher mortality than that predicted by scores in patients with complications of portal hypertension, particularly in patients with acute deterioration of liver function, such as in ACLF. As our databases do not contain systematic data on the specific causes of death, further analyses using data from randomized trials will need to address the question of whether suPAR levels are indicative of specific causes of death such as the development of infections or progression to ACLF, specifically in patients receiving TIPS.

Systemic inflammation is increasingly recognized as a potentially prognostic mechanism following TIPS implantation, particularly in refractory ascites.[23] Several biomarkers of inflammation, such as the chemokines CXCL9[24] and CXCL11,[25] and the soluble TNF receptor,[26,27] have been investigated as prognostic indicators associated with post-TIPS outcomes. However, the independent contribution of more commonly available biomarkers of inflammation, such as WBC or CRP levels, has rarely been confirmed,[28] or only in specific subgroups of patients, such as those with renal failure.[29] Our study is the first to demonstrate that concentrations of a single parameter (suPAR) nominally outperform most other parameters typically used in clinical routine. This finding opens the door for further investigation into more precise risk stratification using a new group of biomarkers that are increasingly being used for risk prediction in cirrhosis, such as macrophage activation or endothelial damage markers.

Nevertheless, we acknowledge important limitations of our study, most of which are unavoidable due to the retrospective study design with a large share of patients receiving uncovered stents for preventing recurrent bleeding, where the survival benefit of TIPS is uncertain. Due to the retrospective study design, a full homogenization with respect to a detailed assessment of clinical factors such as the evolution of comorbidities including heart failure was not feasible. In addition, the cohort sizes were too small for a detailed assessment of individual subgroups, such as the indication for TIPS, and the Child-Pugh subgroup analyses presented are exploratory in nature. As "routine" patients were included, risk assessment for TIPS was based on classical scores that correlate with suPAR, potentially introducing a bias into the analyses. In addition, the etiology of most patients was alcohol-related liver disease, but ongoing alcohol consumption was not routinely assessed. Therefore, the effect of ongoing alcohol abuse on suPAR levels cannot be assessed. The extended recruitment period between the two cohorts leads to heterogeneity with respect to the implanted stents ("Wallstent" *vs.* "VIATORR" stents) and possibly to a measurement imprecision in the longer-stored samples. This may also explain the differences in outcome that we observed between the training and validation cohorts. Most importantly, the use of HV or PV blood for the risk assessment prior to TIPS placement is not feasible in clinical routine (except for HV blood measurements during a potential invasive HVPG measurement).

Elevated circulating suPAR levels in cubital vein blood were predictive of higher mortality risk after TIPS implantation, even in patients classified as low risk by conventional criteria (elective placement, Child-Pugh A/B, low MELD score, and bilirubin levels). Therefore, incorporating venous suPAR measurement into pre-TIPS evaluations could enhance the detection of high-risk patients who might benefit from closer monitoring post implantation. Further studies focusing on peripheral blood are needed for correlation and validation before suPAR measurements can be incorporated into clinical patient management. Along this line of thinking, longitudinal measurements of suPAR following TIPS implantation might yield further prognostic information.[23]

In conclusion, our data provide strong evidence that venous suPAR levels are a novel prognostic marker in patients undergoing TIPS and may help to identify ideal candidates for this increasingly relevant therapy. suPAR concentrations were predictive of patients' prognosis and identified a subgroup of patients who may particularly benefit from TIPS. If these can be confirmed in further longitudinal clinical trials using independent cohorts, our results may open the door to the potential clinical use of circulating suPAR as a non-invasive risk prediction tool in this challenging clinical setting.

Abbreviations

ACLF, acute-on-chronic liver failure; CRP, C-reactive protein; HR, hazard ratio; HRS, hepatorenal syndrome; HV, hepatic vein; HVPG, hepatic venous pressure gradient; MELD, model for end-stage liver disease; OS, overall survival; PV, portal vein; ROC, receiver-operating characteristic; suPAR, soluble urokinase plasminogen activator receptor; TIPS, transjugular intrahepatic portosystemic shunt; WBC, white blood cell.

Financial support

J.T. was supported by the German Research Foundation (DFG) project ID 403224013 – SFB 1382 (A09), by the German Federal Ministry of Education and Research (BMBF) for the DEEP-HCC project and by the Hessian Ministry of Higher Education, Research and the Arts (HMWK) for the ENABLE and ACLF-I cluster projects. The MICROB-PREDICT (project ID 825694), DECISION (project ID 847949), GALAXY (project ID 668031), LIVERHOPE (project ID 731875), and IHMCSA (project ID 964590) projects have received funding from the European Union's Horizon 2020 research and innovation program. The manuscript reflects only the authors' views, and the European Commission is not responsible for any use that may be made of the information it contains. The funders had no influence on study design, data collection and analysis, decision to publish, or preparation of the manuscript. T.L. is supported by the European Research Council (ERC) under the European Union's Horizon 2020 research and innovation program through the ERC Consolidator Grant PhaseControl (Grant Agreement 771083). The laboratory of T. L. was further funded by the German Cancer Aid (Deutsche Krebshilfe – 110043), the Deutsche Forschungsgemeinschaft (DFG, German Research Foundation) – 403224013, 279874820, 461704932, 440603844, the German Ministry of Health (BMG – DEEP LIVER 2520DAT111) and support from the Medical Faculty of the Heinrich Heine University. F.T. is supported by the German Research Foundation (DFG SFB/TRR 296 and CRC1382, Project-ID 403224013) and the German Ministry of Education and Research (BMBF DEEP-HCC consortium, BMBF Immun Avatar consortium). T.B. received funding from the German Research Foundation (SFB1382 Project ID 403224013/B07). C.R. received funding from the German Research Foundation (DFG RO 4317/4-1) and support from the Medical Faculty of the Heinrich Heine University.

Conflict of interest

The authors declare that they have no competing interests related to this manuscript. S.H.L. has received honoraria for consulting or lectures from BMS and MSD.

PA.R received lecture and consulting fees from CSL Behring, Pfizer, Gilead and BristolMyersQuipp and Boston Scientific. F.T.'s lab has received research funding from Allergan, Bristol-Myers Squibb, Gilead and Inventiva. F.T. has received honoraria for consulting or lectures from Astra Zeneca, Gilead, AbbVie, BMS, Boehringer, Madrigal, Intercept, Falk, Ionis, Inventiva, Merz, Pfizer, Alnylam, NGM, CSL Behring, Novo Nordisk, Novartis. T.B. received consulting fees from Intercept/Advanz Pharma, Grifols, and Sobi as well as honoraria for lectures, presentations, or educational events from Falk Foundation, CSL Behring, Merck, Gilead, Intercept/Advanz Pharma, and Gore. J.T. has received speaking and/or consulting fees from Versantis, Gore, Boehringer-Ingelheim, Falk, Grifols, Genfit and CSL Behring. C.R.ás lab has received research funding from Servier and LamKap Bio. C.R. has received honoraria for consulting or lectures from Amgen, Astra Zeneca, BMS, Deciphera, Esteve, Incyte, Ipsen, Novartis, MSD, Merck, Lilly, Pierre Fabre, Roche, Falk, Servier.

Please refer to the accompanying ICMJE disclosure forms for further details.

Authors' contributions

FB, FT, JT, SHL and CR designed the study. LJ and JR performed suPAR measurements. FB, SHL, CR and TB performed statistical analyses. THW, JT, RS and PAR collected data and organized patient recruitment. CR, SHL, FB and TB wrote the manuscript. SHL, CR, JT, CT and TB revised the manuscript for important intellectual content. All other authors provided intellectual input. All authors approved the final version of the manuscript.

Data availability statement

Data are available from the corresponding author upon meaningful request.

Acknowledgements

The authors would like to express their gratitude to members of the Roderburg and the Tacke lab for the helpful discussions.

Supplementary data

Supplementary data to this article can be found online at https://doi.org/10.1016/j.jhepr.2024.101054.

References

Author names in bold designate shared co-first authorship

[1] **Nicoară-Farcău O**, **Han G**, Rudler M, et al. Effects of early placement of transjugular portosystemic shunts in patients with high-risk acute variceal bleeding: a meta-analysis of individual patient data. Gastroenterology 2021;160:193–205.e10. https://doi.org/10.1053/J.GASTRO.2020.09.026.

[2] Bureau C, Thabut D, Oberti F, et al. Transjugular intrahepatic portosystemic shunts with covered stents increase transplant-free survival of patients with cirrhosis and recurrent ascites. Gastroenterology 2017;152:157–163. https://doi.org/10.1053/J.GASTRO.2016.09.016.

[3] Salerno F, Cammà C, Enea M, et al. Transjugular intrahepatic portosystemic shunt for refractory ascites: a meta-analysis of individual patient data. Gastroenterology 2007;133:825–834. https://doi.org/10.1053/J.GASTRO.2007.06.020.

[4] Trebicka J, Fernandez J, Papp M, et al. The PREDICT study uncovers three clinical courses of acutely decompensated cirrhosis that have distinct pathophysiology. J Hepatol 2020;73:842–854. https://doi.org/10.1016/j.jhep.2020.06.013.

[5] Engelmann C, Clària J, Szabo G, et al. Pathophysiology of decompensated cirrhosis: portal hypertension, circulatory dysfunction, inflammation, metabolism and mitochondrial dysfunction. J Hepatol 2021;75:S49–S66. https://doi.org/10.1016/j.jhep.2021.01.002.

[6] **Jansen C**, **Möller P**, Meyer C, et al. Increase in liver stiffness after transjugular intrahepatic portosystemic shunt is associated with inflammation and predicts mortality. Hepatology 2018;67:1472–1484. https://doi.org/10.1002/hep.29612.

[7] Trebicka J, Amoros A, Pitarch C, et al. Addressing profiles of systemic inflammation across the different clinical phenotypes of acutely decompensated cirrhosis. Front Immunol 2019;10. https://doi.org/10.3389/fimmu.2019.00476.

[8] Thunø M, Macho B, Eugen-Olsen J. suPAR: the molecular crystal ball. Dis Markers 2009;27:157–172. https://doi.org/10.3233/DMA-2009-0657.

[9] Xue A, Xue M, Jackson C, et al. Suppression of urokinase plasminogen activator receptor inhibits proliferation and migration of pancreatic adenocarcinoma cells via regulation of ERK/p38 signaling. Int J Biochem Cel Biol 2009;41:1731–1738. https://doi.org/10.1016/j.biocel.2009.03.004.

[10] Diederichsen MZ, Diederichsen SZ, Mickley H, et al. Prognostic value of suPAR and hs-CRP on cardiovascular disease. Atherosclerosis 2018;271:245–251. https://doi.org/10.1016/J.ATHEROSCLEROSIS.2018.01.029.

[11] Loosen SH, Tacke F, Püthe N, et al. High baseline soluble urokinase plasminogen activator receptor (suPAR) serum levels indicate adverse outcome after resection of pancreatic adenocarcinoma. Carcinogenesis 2019;40:947–955. https://doi.org/10.1093/CARCIN/BGZ033.

[12] Loosen SH, Breuer A, Tacke F, et al. Circulating levels of soluble urokinase plasminogen activator receptor predict outcome after resection of biliary tract cancer. JHEP Rep 2020;2. https://doi.org/10.1016/J.JHEPR.2020.100080.

[13] **Zimmermann HW**, **Koch A**, Seidler S, et al. Circulating soluble urokinase plasminogen activator is elevated in patients with chronic liver disease, discriminates stage and aetiology of cirrhosis and predicts prognosis. Liver Int 2011;32. https://doi.org/10.1111/j.1478-3231.2011.02665.x.

[14] Zimmermann HW, Reuken PA, Koch A, et al. Soluble urokinase plasminogen activator receptor is compartmentally regulated in decompensated cirrhosis and indicates immune activation and short-term mortality. J Intern Med 2013;274:86–100. https://doi.org/10.1111/JOIM.12054.

[15] Berres ML, Schlosser B, Berg T, et al. Soluble urokinase plasminogen activator receptor is associated with progressive liver fibrosis in hepatitis C infection. J Clin Gastroenterol 2012;46:334–338. https://doi.org/10.1097/MCG.0B013E31822DA19D.

[16] **Koch A**, **Zimmermann HW**, Gassler N, et al. Clinical relevance and cellular source of elevated soluble urokinase plasminogen activator receptor (suPAR) in acute liver failure. Liver Int 2014;34:1330–1339. https://doi.org/10.1111/LIV.12512.

[17] Bettinger D, Sturm L, Pfaff L, et al. Refining prediction of survival after TIPS with the novel Freiburg index of post-TIPS survival. J Hepatol 2021;74:1362–1372. https://doi.org/10.1016/j.jhep.2021.01.023.

[18] **Arroyo V**, **Angeli P**, **Moreau R**, et al. The systemic inflammation hypothesis: towards a new paradigm of acute decompensation and multiorgan failure in cirrhosis. J Hepatol 2021;74:670–685. https://doi.org/10.1016/J.JHEP.2020.11.048.

[19] **Costa D**, **Simbrunner B**, Jachs M, et al. Systemic inflammation increases across distinct stages of advanced chronic liver disease and correlates

with decompensation and mortality. J Hepatol 2021;74:819–828. https://doi.org/10.1016/J.JHEP.2020.10.004.
[20] **Jalan R**, **Saliba F**, **Pavesi M**, et al. Development and validation of a prognostic score to predict mortality in patients with acute-on-chronic liver failure. J Hepatol 2014;61:1038–1047. https://doi.org/10.1016/J.JHEP.2014.06.012.
[21] Wirtz TH, Reuken PA, Jansen C, et al. Balance between macrophage migration inhibitory factor and sCD74 predicts outcome in patients with acute decompensation of cirrhosis. JHEP Rep 2020;3. https://doi.org/10.1016/J.JHEPR.2020.100221.
[22] Jalan R, Pavesi M, Saliba F, et al. The CLIF Consortium Acute Decompensation score (CLIF-C ADs) for prognosis of hospitalised cirrhotic patients without acute-on-chronic liver failure. J Hepatol 2015;62:831–840. https://doi.org/10.1016/J.JHEP.2014.11.012.
[23] **Deltenre P**, **Zanetto A**, Saltini D, et al. The role of transjugular intrahepatic portosystemic shunt in patients with cirrhosis and ascites: recent evolution and open questions. Hepatology 2023;77. https://doi.org/10.1002/HEP.32596.
[24] **Berres ML**, **Asmacher S**, Lehmann J, et al. CXCL9 is a prognostic marker in patients with liver cirrhosis receiving transjugular intrahepatic portosystemic shunt. J Hepatol 2015;62:332–339. https://doi.org/10.1016/J.JHEP.2014.09.032.
[25] **Berres ML**, **Lehmann J**, Jansen C, et al. Chemokine (C-X-C motif) ligand 11 levels predict survival in cirrhotic patients with transjugular intrahepatic portosystemic shunt. Liver Int 2016;36:386–394. https://doi.org/10.1111/LIV.12922.
[26] Trebicka J, Krag A, Gansweid S, et al. Endotoxin and tumor necrosis factor-receptor levels in portal and hepatic vein of patients with alcoholic liver cirrhosis receiving elective transjugular intrahepatic portosystemic shunt. Eur J Gastroenterol Hepatol 2011;23:1218–1225. https://doi.org/10.1097/MEG.0b013e32834a75dc.
[27] Trebicka J, Krag A, Gansweid S, et al. Soluble TNF-alpha-receptors I are prognostic markers in TIPS-treated patients with cirrhosis and portal hypertension. PLoS One 2013;8. https://doi.org/10.1371/journal.pone.0083341.
[28] Keimburg SA, Theysohn J, Buechter M, et al. FIB-4 and APRI as predictive factors for short- and long-term survival in patients with transjugular intrahepatic portosystemic stent shunts. Biomedicines 2022;10. https://doi.org/10.3390/BIOMEDICINES10051018.
[29] Fürschuß L, Rainer F, Effenberger M, et al. A novel score predicts mortality after transjugular intrahepatic portosystemic shunt: MOTS - modified TIPS Score. Liver Int 2022;42:1849–1860. https://doi.org/10.1111/LIV.15236.

Journal of Hepatology, Volume *6*

Supplemental information

Soluble urokinase plasminogen activator receptor levels predict survival in patients with portal hypertension undergoing TIPS

Sven H. Loosen, Fabian Benz, Raphael Mohr, Philipp A. Reuken, Theresa H. Wirtz, Lioba Junker, Christian Jansen, Carsten Meyer, Michael Praktiknjo, Alexander Wree, Johanna Reißing, Münevver Demir, Wenyi Gu, Mihael Vucur, Robert Schierwagen, Andreas Stallmach, Anselm Kunstein, Johannes Bode, Christian Trautwein, Frank Tacke, Tom Luedde, Tony Bruns, Jonel Trebicka, and Christoph Roderburg

Soluble urokinase plasminogen activator receptor levels predict survival in patients with portal hypertension undergoing TIPS

Sven H. Loosen, Fabian Benz, Raphael Mohr, Philipp A. Reuken, Theresa H. Wirtz, Lioba Junker, Christian Jansen, Carsten Meyer, Michael Praktiknjo, Alexander Wree, Johanna Reißing, Münevver Demir, Mihael Vucur, Robert Schierwagen, Andreas Stallmach, Anselm Kunstein, Johannes Bode, Christian Trautwein, Frank Tacke, Tom Luedde, Tony Bruns, Jonel Trebicka, Christoph Roderburg

Table of contents

Supplementary Figures……………………………………………………………………..2

Supplementary Tables…………………………………………………………………………8

Supplementary Figures

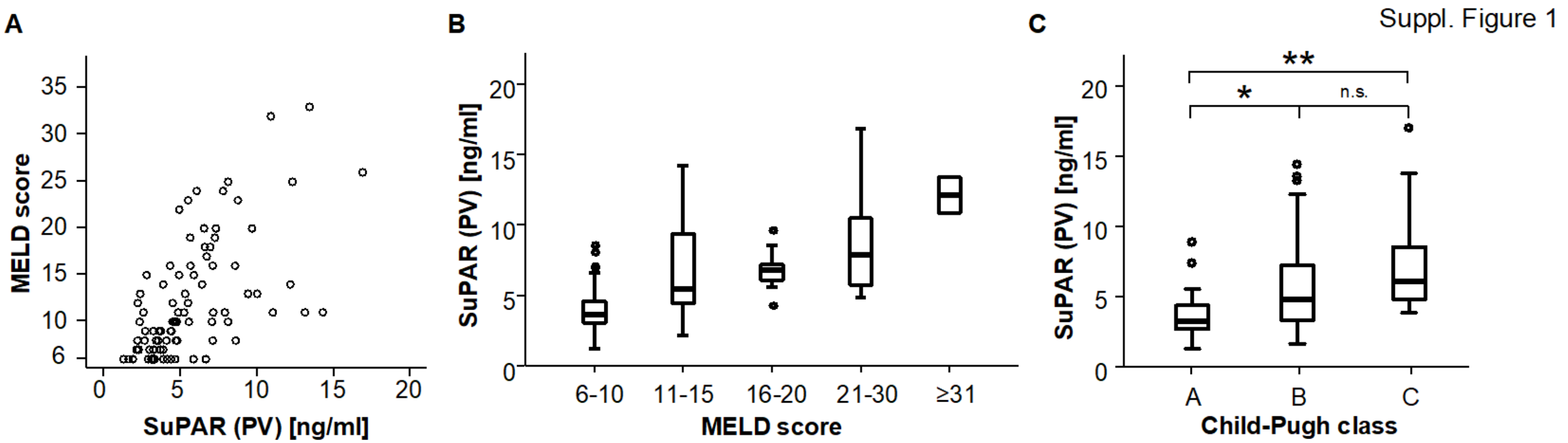

Fig. S1: (A) Portal vein (PV) suPAR levels correlate with the patients' MELD score. (B) PV suPAR concentrations show a stepwise increase in patients with more advanced MELD score. (C) PV suPAR concentrations show a significant and stepwise increase in patients with more advanced Child-Pugh class.

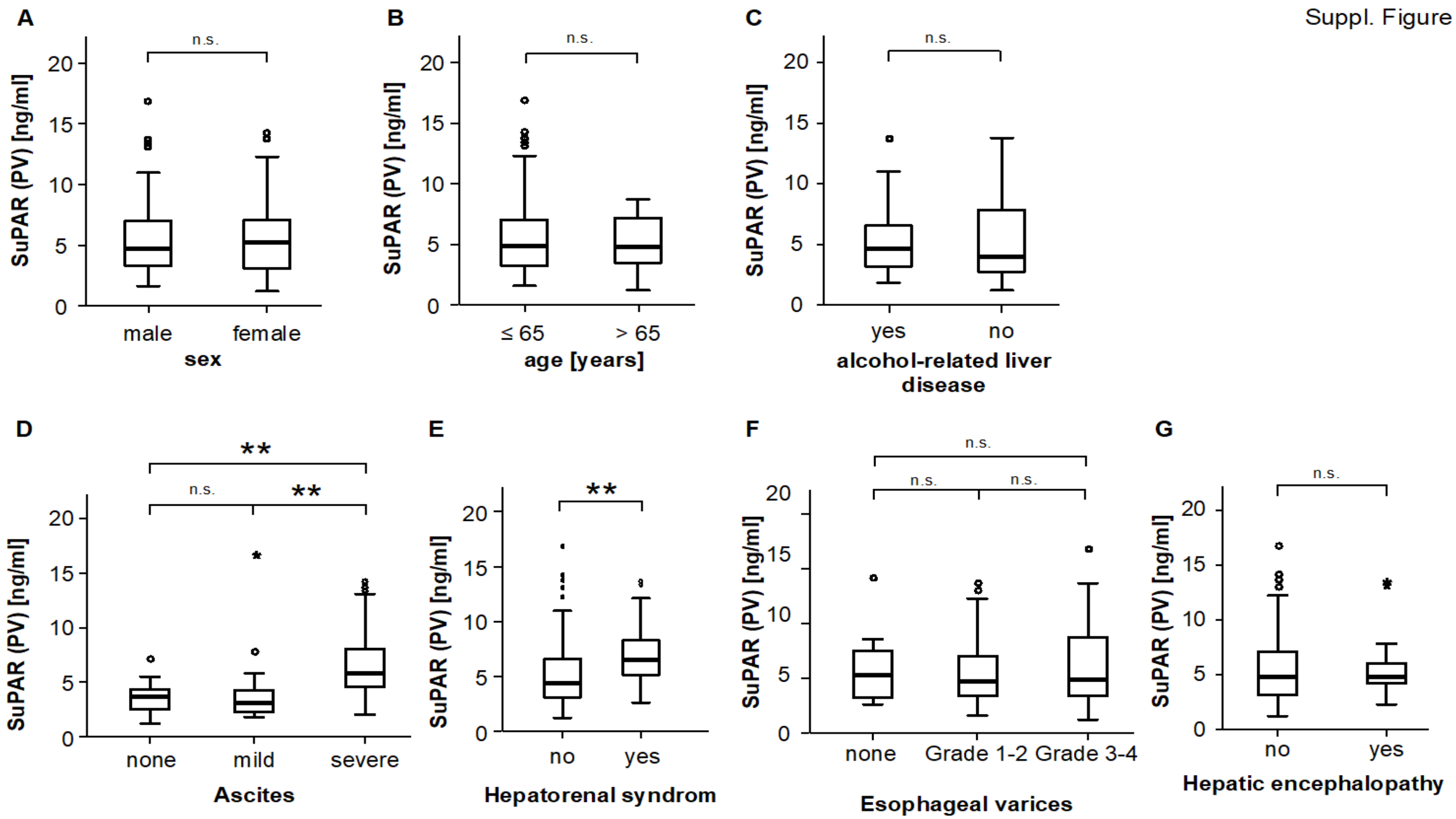

Fig. S2: (A-C) Hepatic vein (HV) suPAR levels are comparable in male and female patients, in patients younger or older than 65 years, and in patients with or without alcohol-related liver disease. (D) HV suPAR levels are significantly higher among patients with moderate to severe ascites compared to patients without or mild ascites. (E) HV suPAR levels are significantly higher in patients with hepatorenal syndrome (HRS) compared to patients with normal renal function. (F and G) Patients with or without esophageal varices or hepatic encephalopathy have comparable HV suPAR concentrations.

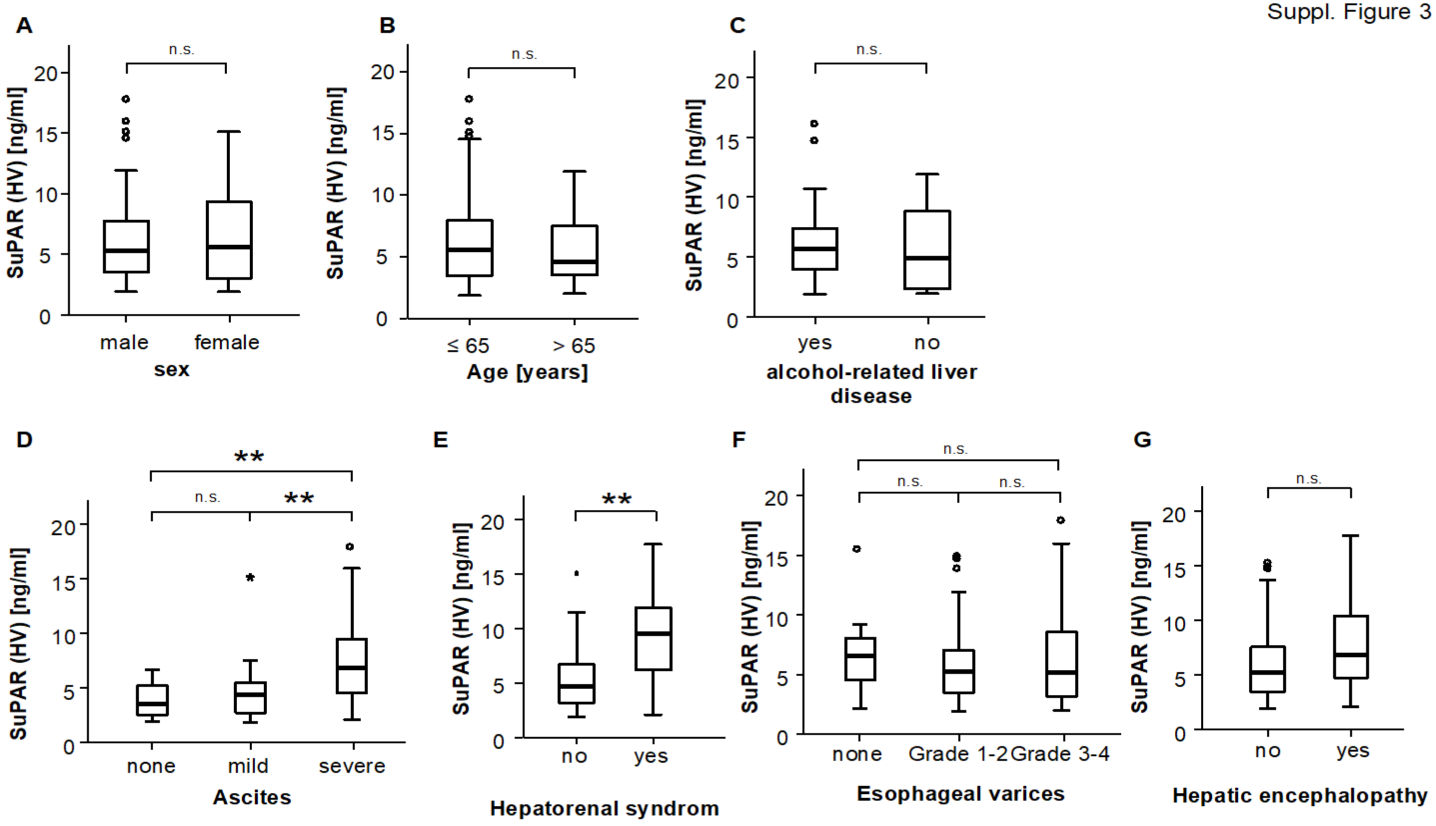

Fig. S3: (A-C) Portal vein (PV) suPAR levels are comparable in male and female patients, in patients younger or older than 65 years, and in patients with or without alcohol-related liver disease. (D) PV suPAR levels are significantly higher among patients with moderate to severe ascites compared to patients without or mild ascites. (E) PV suPAR levels are significantly higher in patients with hepatorenal syndrome (HRS) compared to patients with normal renal function. (F and G) Patients with or without esophageal varices or hepatic encephalopathy have comparable PV suPAR concentrations.

Supp. Figure 4

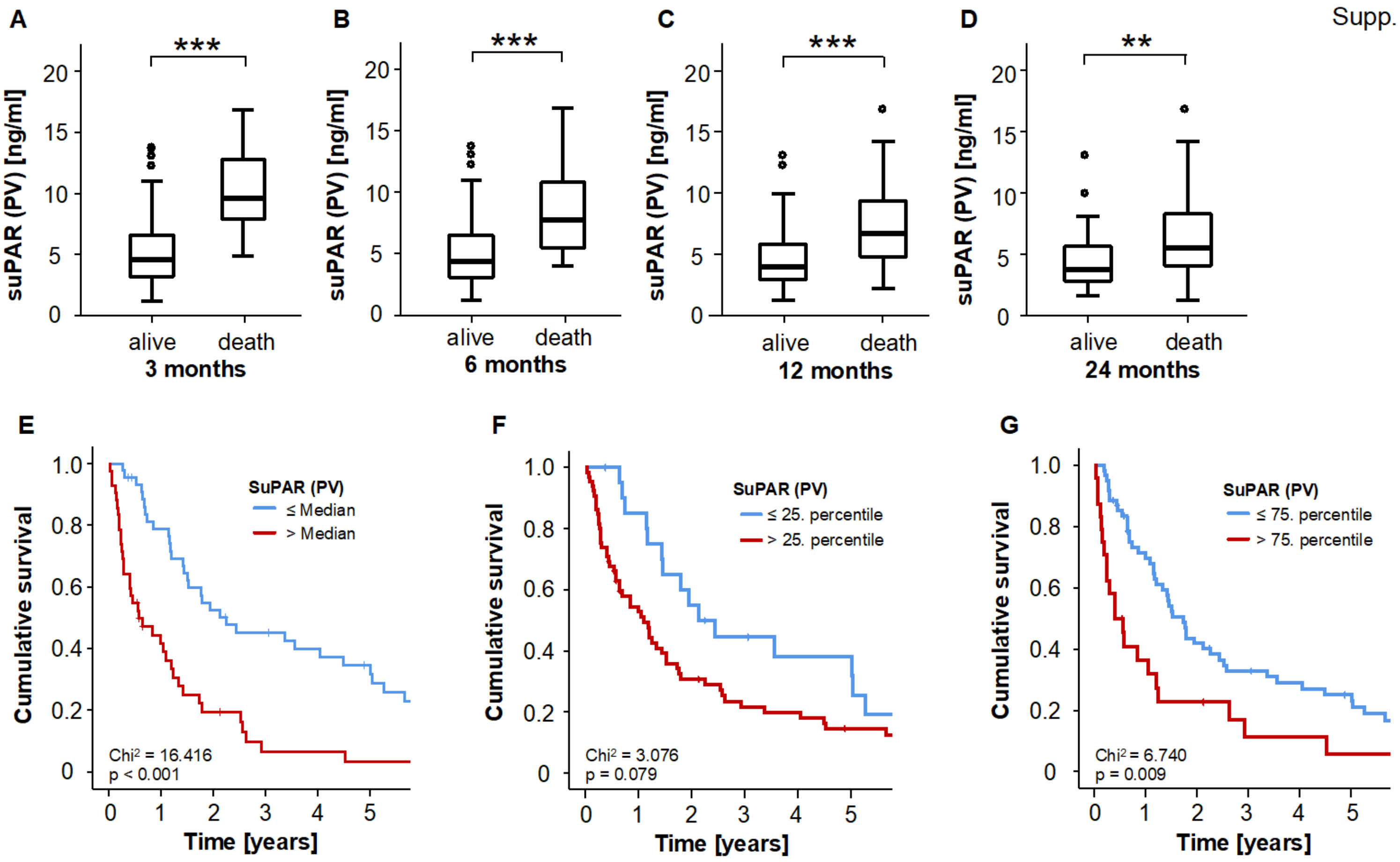

Fig. S4: Portal vein (PV) suPAR levels are significantly higher in patients who died within 3 (A), 6 (B), 12 (C) or 24 months (D) after TIPS insertion. The median OS is reduced in TIPS patients with baseline PV suPAR concentrations above the median (E) or the lower/upper quartile (F, G).

Suppl Figure 5

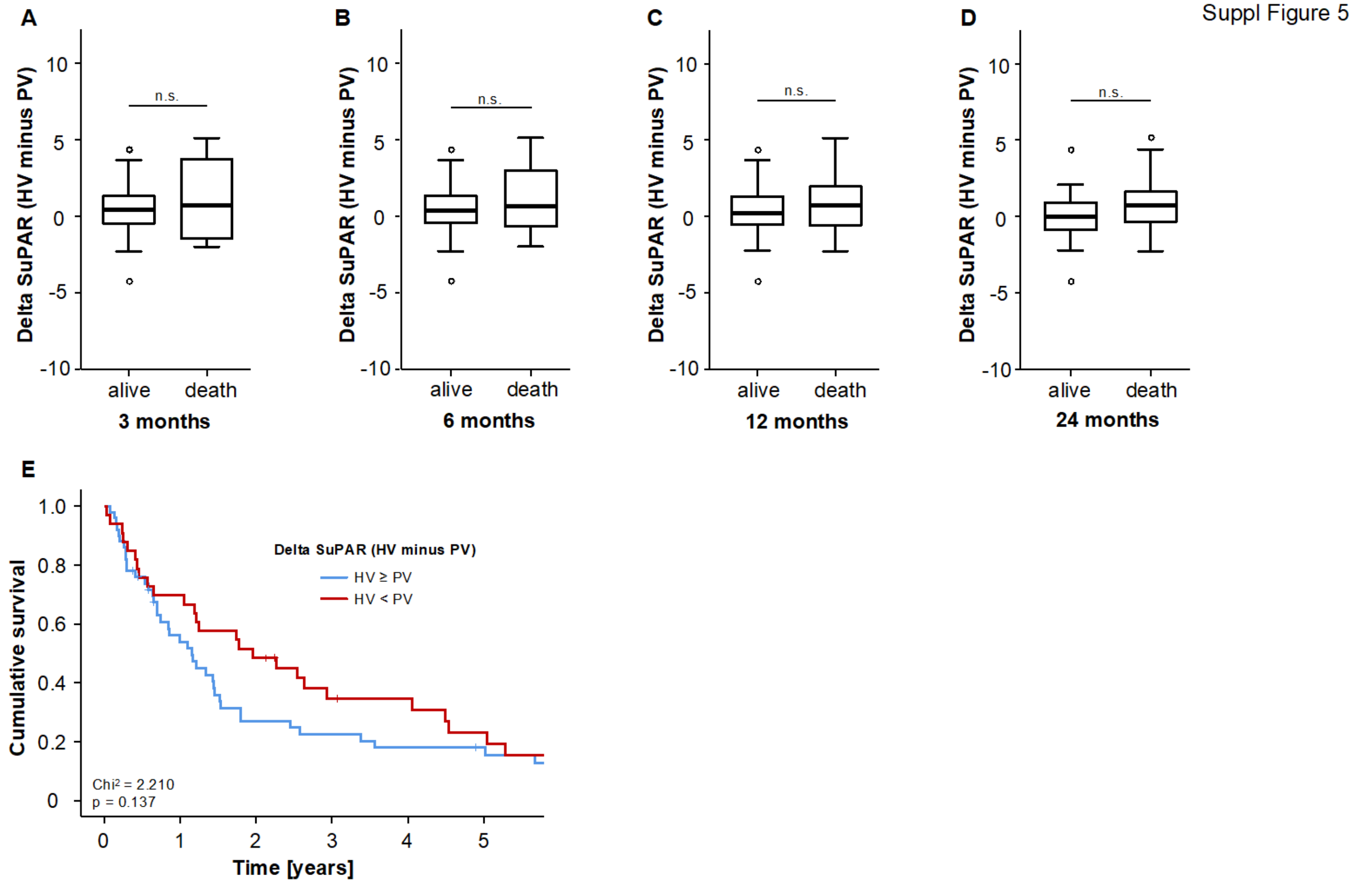

Fig. S5: (A-D) The individual ratio between HV and PV suPAR concentrations (delta suPAR = HV-PV) is unaltered TIPS patients who did or did not survive the 3-, 6-, 12-, or 24-months period following TIPS insertion. (E) There is no survival benefit for TIPS patients with either a positive or negative delta suPAR.

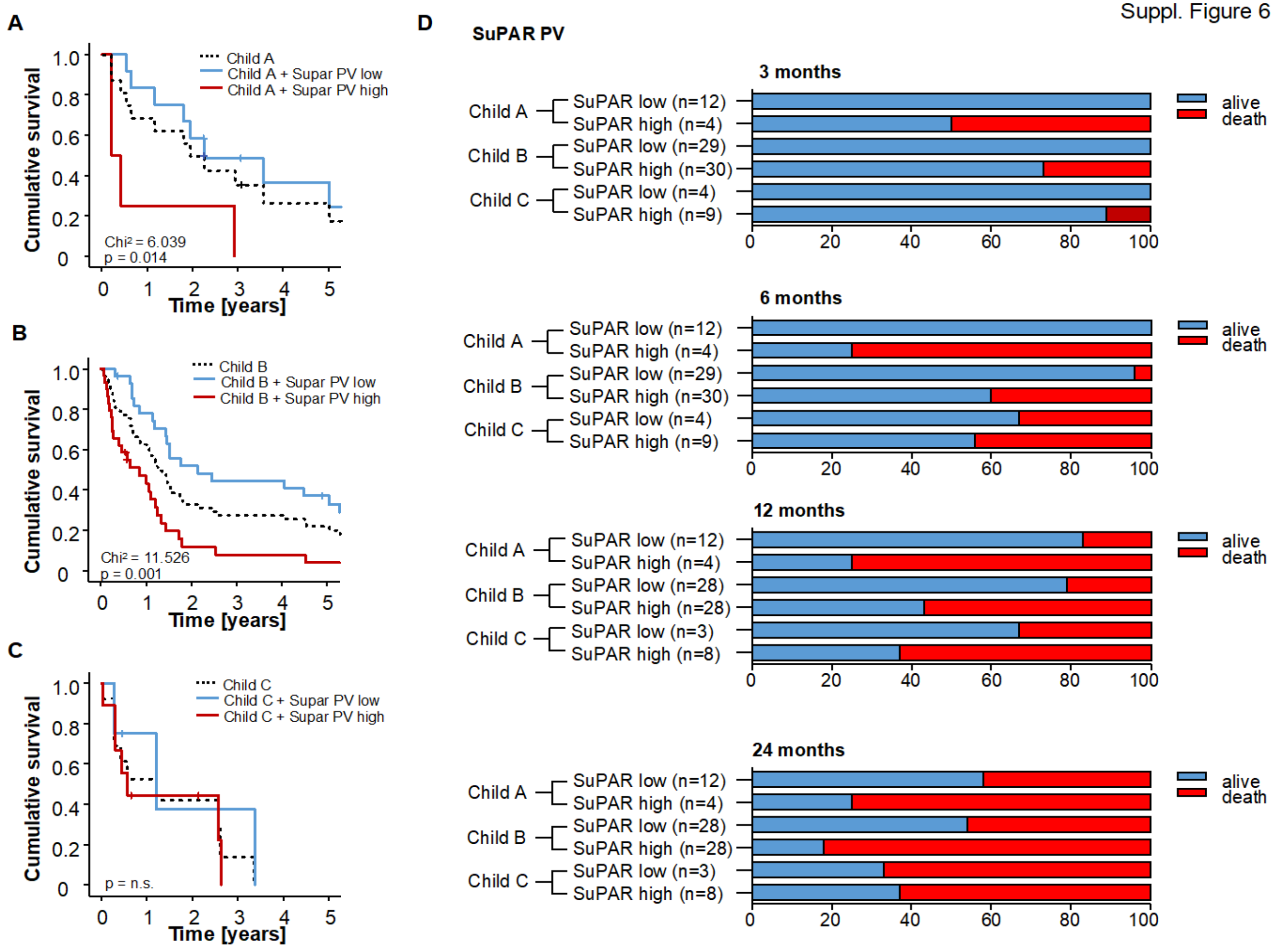

Fig. S6: (A) In Child A patients, elevated PV suPAR levels identify a subgroup of TIPS patients with a significantly impaired post-interventional median overall survival, which is lower compared to patients with Child B and Child C cirrhosis. (B) In Child B patients, elevated PV suPAR levels identify a subgroup of TIPS patients with a significantly impaired post-interventional outcome. (C) This finding is not observed in Child C patients. (D) Number of Child-Pugh stage-stratified deaths after 3, 6, 12, and 24 months in TIPS patient with high or low baseline PV suPAR levels.

Supplementary Tables

Table S1. Clinical and laboratory parameters of the training cohort.

	SuPAR serum concentration[ng/ml]		
**Parameters**	**Hepatic vein**	**Portal vein**	**p-value**
All patients (n=99)	5.27 (1.90-17.76)	4.76 (1.22-16.84)	p<0.01
Sex			
male: 67 %	5.27 (1.90-17.76)	4.70 (1.61-16.84)	p<0.05
female: 33 %	5.56 (1.92-15.10)	5.26 (1.22-14.23)	p<0.05
Age: 59 years (36-77)			
≤ 65 years: 78 %	5.56 (1.90-17.76)	4.85 (1.58-16.84)	p<0.05
> 65 years: 22 %	4.65 (2.05-11.91)	4.79 (1.22-8.70)	n.s.
BMI: 24.5 kg/m^2 (15.2-38.9)			
<20: 16 %	5.10 (1.92-11.46)	4.96 (1.58-13.75)	n.s.
20-25: 41 %	6.04 (1.99-14.56)	4.79 (1.22-10.85)	p<0.05
>25: 43 %	5.08 (1.90-15.96)	4.19 (1.83-13.67)	p<0.05
Etiology			
alcoholic: 73 %	5.70 (1.90-15.96)	3.95 (1.22-13.75)	p<0.05
non-alcoholic: 27 %	4.91 (1.92-11.91)	4.96 (1.58-13.75)	n.s.
Ascites			
no: 19 %	3.56 (1.92-6.65)	3.65 (1.22-7.19)	n.s.
mild: 17 %	4.39 (1.90-15.10)	3.09 (1.83-16.84)	p<0.05
moderate to severe: 64 %	6.86 (2.11-17.76)	5.81 (2.03-14.23)	p<0.05
History of hepatic encephalopathy			
no: 85 %	5.27 (1.90-15.10)	4.79 (1.22-16.84)	p<0.05
yes: 15 %	6.16 (2.07-17.76)	4.79 (2.28-13.67)	p<0.05
Hepatorenal syndrome			
no: 77 %	4.70 (1.90-15.10)	4.35 (1.22-16.84)	p<0.05
yes: 23 %	9.54 (2.11-17.76)	6.54 (2.61-13.67)	p<0.05
Esophageal varices			
no: 12 %	6.58 (2.11-15.10)	5.26 (2.61-14.23)	p<0.05
grade I-II: 66 %	5.27 (1.90-14.76)	4.70 (1.58-13.75)	p<0.05
grade III-IV: 22 %	5.15 (1.99-17.76)	4.86 (1.22-16.84)	n.s.
Child category			
Child A: 17 %	2.92 (1.92-9.54)	3.19 (1.22-8.70)	n.s.
Child B: 67 %	5.25 (1.90-17.76)	4.79 (1.61-14.23)	p<0.05
Child C: 16 %	7.34 (4.50-15.96)	6.04 (3.81-16.84)	p<0.05
MELD score: 10 (6-33)			
6-10: 52 %	4.39 (1.90-10.69)	3.67 (1.22-8.56)	p<0.05
11-15: 24 %	5.59 (2.60-15.10)	5.45 (2.16-14.23)	n.s.
16-20: 13 %	7.06 (4.50-14.76)	6.78 (4.27-9.62)	n.s.
21-30: 9%	9.96 (4.47-15.10)	7.89 (4.87-16.84)	n.s.
>30: 2%	16.16 (14.56-17.76)	12.11 (10.85-13.37)	n.s.

Data are shown as median and ranges

Table S2. Laboratory parameters (hepatic vein) of the training cohort.

**Laboratory parameters**	**Value**
Sodium	135 mmol/l (119-143)
Potassium	4.2 mmol/l (2.6-5.8)
Creatinine	1.1 mg/dl (0.5-8.2)
Urea	44 mg/dl (9-225)
Bilirubin	1.3 mg/dl (0.4-16.9)
Albumin	32 g/dl (11-56)
International Normalized Ratio (INR)	1.13 (0.95-2.40)
Partial thromboplastin time (PTT)	28 seconds (22-100)
Thrombocyte count	104 /nl (27-389)
Leukocyte count	5.5 /nl (1.4-22.3)
Glutamat-Oxalacetat-Transaminase (GOT)	21 U/l (8-73)
Glutamat-Pyruvat-Transaminase (GPT)	18 U/l (4-113)
Gamma-Glutamyltransferase (GGT)	57 U/l (8-1469)
Cholinesterase (ChE)	1804 U/l (282-11937)
Ammonia (NH3)	47 µmol/l (4-137)

Data are shown as median and ranges

Table S3. Hemodynamic parameters of the training cohort before TIPS.
Data are shown as median and ranges

Hemodynamic parameters before TIPS	Values
Mean arterial pressure	84 mmHg (48-129)
Central venous pressure	10 cmH2O (0-22)
Portal pressure	30 mmHg (18-49)
Hepatic venous pressure gradient (HVPG)	20 mmHg (11-35)
Portal venous velocity	16 (5-40)
Portal venous area	1505 (510-4320)

Table S4. SuPAR level with respect to the survival status (training cohort)

	SuPAR level Hepatic vein (HV)	SuPAR level Portal vein (PV)
3-month survival		
yes: 88 %	5.08 (1.90-15.96)	4.60 (1.22-13.75)
no: 12 %	14.14 (5.27-17.76)	9.62 (4.85-16.84)
6-month survival		
yes: 76 %	4.73 (1.90-11.91)	4.36 (1.22-13.75)
no: 24 %	8.77 (3.68-17.76)	7.72 (4.04-16.84)
12-month survival		
yes: 61 %	4.64 (1.90-11.28)	3.95 (1.22-13.08)
no: 39 %	7.06 (3.13-17.76)	6.66 (2.16-16.84)
24-month survival		
yes: 37 %	3.44 (1.90-9.16)	3.75 (1.58-13.08)
no: 63 %	6.14 (1.99-17.76)	5.50 (1.22-16.84)

Data are shown as median and ranges

Table S5: Comparison of patient characteristics between patients with high or low suPAR levels (median)

Parameter	Training cohort			Validation cohort		
	**HV suPAR < median (5.27ng/ml)**	**HV suPAR > median (5.27ng/ml)**	**p-value**	**suPAR < median (8.93 ng/ml)**	**suPAR > median (8.93 ng/ml)**	**p-value**
**Age** (years, median, IQR)	59.5 (15)	59.0 (12)	0.706	61 (13)	57 (12)	0.122
**BMI** (kg/m^2, median, IQR)	25.65 (5.23)	24.02 (5.27)	0.354	N/A*	N/A*	-
**Sex** (%)						
male	68.2	66.0		77.3	76.0	
female	31.8	34.0	0.822	22.7	24.0	1.000
**TIPS indication** (%)						
Ascites	27.7	60.9		88.0	81.3	
Variceal bleeding	53.2	26.1		12.0	16.0	
Variceal bleeding and ascites	8.5	10.8		0.0	2.7	
HRS	10.6	2.2	0.005	0.0	0.0	0.320
**Child-Pugh** (%)						
Class A	25.5	8.7		8.0	4.0	
Class B	70.2	60.7		69.3	60.0	
Class C	4.3	30.4	0.001	22.7	36.0	0.140
**Etiology** (%)						
Alcohol-related	70.7	80.4		73.3	86.7	
NAFLD	-	-		9.3	5.3	
Cholestatic liver disease	2.4	4.3		2.7	2.7	
Viral	9.8	13.0		2.7	0.0	
Other	17.1	2.2	0.182	12.0	5.3	0.250

*As the majority of patients had tense ascites, BMI was not assessed in the validation cohort.

Table S6: Uni- and multivariate Cox-regression analyses for overall survival including the MELD-Na score (training cohort)

	Univariate regression models		Multivariate regression model	
**Parameter**	**p-value**	**Hazard ratio (CI)**	**p-value**	**Hazard ratio (CI)**
suPAR HVs	<0.001	1.299 (1.198-1.409)	0.001	1.215 (1.085-1.359)
MELD-Na	<0.001	1.112 (1.069-1.156)	0.029	1.057 (1.006-1.111)
Potassium	0.034	1.434 (1.028-2.000)	0.049	1.404 (1.002-1.968)

Table S7: Uni- and multivariate Cox-regression analyses for overall survival including the FIPS score (training cohort)

	Univariate regression models		Multivariate regression model	
**Parameter**	**p-value**	**Hazard ratio (CI)**	**p-value**	**Hazard ratio (CI)**
suPAR HV	<0.001	1.299 (1.198-1.409)	<0.001	1.247 (1.127-1.380)
Sodium	0.004	0.927 (0.881-0.976)	0.408	0.977 (0.923-1.033)
Potassium	0.034	1.434 (1.028-2.000)	0.159	1.282 (0.907-1.811)
FIPS score	<0.001	1.888 (1.363-2.617)	0.073	1.401 (0.969-2.027)

FIPS: Freiburg index of post-TIPS survival

Table S8: Uni- and multivariate Cox-regression analyses for transplant-free survival (training cohort)

	**Univariate regression models**		**Multivariate regression model**	
**Parameter**	**p-value**	**Hazard ratio (CI)**	**p-value**	**Hazard ratio (CI)**
suPAR HV	<0.001	1.287 (1.192-1.390)	0.001	1.190 (1.075-1.317)
suPAR PV	<0.001	1.173 (1.097-1.255)		
Creatinine	<0.001	1.660 (1.361-2.025)	0.096	1.251 (0.961-1.628)
INR	0.191	1.686 (0.771-3.688)		
Bilirubin	0.003	1.266 (1.081-1.481)	0.048	1.198 (1.002-1.432)
ALT	0.882	1.001 (0.988-1.014)		
AST	0.370	0.992 (0.973-1.010)		
GGT	0.180	0.999 (0.997-1.001)		
Albumin	0.809	0.996 (0.964-1.029)		
Sodium	0.001	0.919 (0.875-0.965)	0.100	0.959 (0.912-1.008)
Potassium	0.033	1.415 (1.028-1.947)	0.044	1.440 (1.009-2.055)
Leucocyte count	0.049	1.092 (1.000-1.191)	0.876	1.008 (0.911-1.116)
Thrombocyte count	0.511	1.001 (0.998-1.005)		
Age	0.117	1.025 (0.995-1.050)		
BMI	0.752	1.013 (0.935-1.097)		
Sex	0.978	1.007 (0.632-1.628)		
